# Non-host Resistance Induced by the *Xanthomonas* Effector XopQ Is Widespread within the Genus *Nicotiana* and Functionally Depends on EDS1

**DOI:** 10.3389/fpls.2016.01796

**Published:** 2016-11-30

**Authors:** Norman Adlung, Heike Prochaska, Sabine Thieme, Anne Banik, Doreen Blüher, Peter John, Oliver Nagel, Sebastian Schulze, Johannes Gantner, Carolin Delker, Johannes Stuttmann, Ulla Bonas

**Affiliations:** ^1^Department of Genetics, Institute for Biology, Martin Luther University Halle-WittenbergHalle, Germany; ^2^Department of Crop Physiology, Institute of Agricultural and Nutritional Sciences, Martin Luther University Halle-WittenbergHalle, Germany

**Keywords:** Non-host resistance, *Solanaceae*, *Nicotiana benthamiana*, *Xanthomonas*, XopQ, XopC, EDS1, ETI

## Abstract

Most Gram-negative plant pathogenic bacteria translocate effector proteins (T3Es) directly into plant cells via a conserved type III secretion system, which is essential for pathogenicity in susceptible plants. In resistant plants, recognition of some T3Es is mediated by corresponding resistance (*R*) genes or R proteins and induces effector triggered immunity (ETI) that often results in programmed cell death reactions. The identification of *R* genes and understanding their evolution/distribution bears great potential for the generation of resistant crop plants. We focus on T3Es from *Xanthomonas campestris* pv. *vesicatoria* (*Xcv*), the causal agent of bacterial spot disease on pepper and tomato plants. Here, 86 *Solanaceae* lines mainly of the genus *Nicotiana* were screened for phenotypical reactions after *Agrobacterium tumefaciens*-mediated transient expression of 21 different *Xcv* effectors to (i) identify new plant lines for T3E characterization, (ii) analyze conservation/evolution of putative *R* genes and (iii) identify promising plant lines as repertoire for *R* gene isolation. The effectors provoked different reactions on closely related plant lines indicative of a high variability and evolution rate of potential *R* genes. In some cases, putative *R* genes were conserved within a plant species but not within superordinate phylogenetical units. Interestingly, the effector XopQ was recognized by several *Nicotiana* spp. lines, and *Xcv* infection assays revealed that XopQ is a host range determinant in many *Nicotiana* species. Non-host resistance against *Xcv* and XopQ recognition in *N. benthamiana* required *EDS1*, strongly suggesting the presence of a TIR domain-containing XopQ-specific R protein in these plant lines. XopQ is a conserved effector among most xanthomonads, pointing out the XopQ-recognizing R_xopQ_ as candidate for targeted crop improvement.

## Introduction

Plants have evolved different defense mechanisms for protection against potentially pathogenic microbes. As a first line of defense, surface-localized plant receptors recognize pathogen-associated molecular patterns (PAMPs) such as flagellin or lipopolysaccharide and initiate PAMP-triggered immunity, PTI (Jones and Dangl, 2006; Schwessinger and Ronald, 2012). Most Gram-negative plant-pathogenic bacteria express a conserved type III secretion system (T3SS) and translocate type III effector (T3E) proteins directly into the plant cell cytosol (Büttner and He, 2009). Here, T3Es manipulate plant cellular processes in various ways for the benefit of the bacteria, e.g., to suppress PTI (Büttner, 2016). On the other hand, plants can recognize T3Es via resistance (R) genes or proteins that in return initiate effector-triggered immunity, ETI (Khan et al., 2016). PTI and ETI are characterized by different cellular defense mechanisms, i.e., induction of mitogen-activated protein kinases, transcriptional reprogramming, formation of reactive oxygen species and a Ca^2+^-burst (Meng and Zhang, 2013; Buscaill and Rivas, 2014; Cui et al., 2015; Kadota et al., 2015). Most plant R proteins belong to the nucleotide-binding leucine-rich repeat receptors (NLRs), usually harboring an N-terminal coiled-coil domain (CNLs) or a Toll interleukin-1 receptor domain (TNLs) (Li et al., 2015). Typically, NLRs are bound to adenosine diphosphate (ADP) in an inactive state. Presence of a corresponding effector most likely induces a conformational change, leading to the exchange of ADP to adenosine triphosphate (ATP) and ultimately the exposure of the N-terminal domain, which is believed to initiate downstream signaling processes (Takken and Goverse, 2012; Sukarta et al., 2016). NLR specificity is usually conferred by the highly diverse C-terminal LRR domain, and direct effector-binding has been shown in some cases (Li et al., 2015). Alternatively, effectors can be sensed indirectly by NLRs guarding effector targets (guardee) or mimics thereof (decoy) (Khan et al., 2016), and decoys were recently found to also persist integrated into NLRs (Cesari et al., 2014). In some cases, ETI is induced without NLRs. This was shown for transcription activator-like effectors (TALEs), which activate transcription of non-NLR encoding *R* genes (Boch et al., 2014). ETI often results in the hypersensitive response (HR), a rapid programmed cell death limiting bacterial multiplication (Klement and Goodman, 1967).

In contrast to host plant-specific resistance, plant non-host resistance (NHR) is defined as the resistance of all genotypes of an entire plant species to all genotypes of a pathogen species (Gill et al., 2015). NHR is the most common form of plant resistance, directed against a multitude of pathogens (Heath, 2000; Niks and Marcel, 2009; Fan and Doerner, 2012). NHR is complex and includes physical barriers (e.g., the plant cuticle), plant species-specific secondary metabolites which are sufficient to defend poorly adapted pathogens and might include PTI and even ETI mechanisms (Thordal-Christensen, 2003; Maekawa et al., 2011). Plant NHR reactions vary from symptomless reactions to HR (Uma et al., 2011). Non-host plants represent an excellent repertoire of *R* genes and potentially novel resistance mechanisms, which can be employed to generate resistant crop plants (Bent, 2016; Lee et al., 2016).

We study the γ-proteobacterium *Xanthomonas campestris* pv. *vesicatoria* (*Xcv*), the causal agent of bacterial spot disease on pepper and tomato plants which causes enormous yield losses in regions with a warm and humid climate (Stall, 1995). *Xcv* translocates approximately 35 different T3Es into the host cell cytosol (Thieme et al., 2005; Teper et al., 2016). Here, T3Es interfere with plant cellular processes, e.g., via transcriptional reprogramming (Kay et al., 2007; Römer et al., 2007), ubiquitination (Singer et al., 2013), desumoylation (Kim et al., 2013), or modulation of proteasome activity (Üstün et al., 2013), and often suppress PTI (Popov et al., 2016). A helpful tool for T3E characterization is the *Agrobacterium tumefaciens*-mediated transient expression of individual T3Es in model plants of the genus *Nicotiana*, particularly *N. benthamiana* and *N. tabacum* both non-host plants for *Xcv*. Several *Xcv* T3Es induce cell death reactions in *Nicotiana* spp., presumably as a result of ETI upon T3E recognition. For example, transient expression of XopJ (Thieme et al., 2007), XopE1 (Thieme et al., 2007), XopL (Singer et al., 2013), XopX (Metz et al., 2005; Stork et al., 2015), AvrRxv, and AvrBsT (Schulze et al., 2012) induces severe cell death reactions in *N. benthamiana*, whereas expression of XopG induces cell death in *N. tabacum* (Schulze et al., 2012).

To obtain a larger picture on the recognition of *Xcv* effectors in *Solanaceae* spp., we used in this study a set of 21 T3Es, which were transiently expressed in a large panel of plant lines. Our results indicate that T3E families or homologies do not correlate with recognition in different plant lines. Furthermore, assumed *R* genes for recognition of T3Es are highly divergent at all phylogenetic levels. One particular *Xcv* effector, XopQ, was identified as a host range-limiting factor in several *Nicotiana* species, and is most likely recognized by a TIR-type NLR at least in *N. benthamiana*.

## Materials and methods

### Bacterial strains and growth conditions

*Escherichia coli* TOP10 (Thermo Fisher Scientific), DH5α λpir (Ménard et al., 1993) and derivatives were cultivated in LB (lysogeny broth) medium at 37°C. *A. tumefaciens* GV3101(pMP90) (Koncz and Schell, 1986) and derivatives were grown at 30°C in YEB (yeast extract broth) medium, and *Xcv* 85-10 (Thieme et al., 2005), *Xcv* 85-10Δ*xopQ, Xcv* 85-10Δ*xopC*, and *Xcv* 85-10Δ*hrcN* (Lorenz and Büttner, 2009) at 30°C in nutrient yeast glycerol (Daniels et al., 1984). Plasmids were introduced into *E. coli* and *A. tumefaciens* by chemical transformation and electroporation, respectively, and into *Xcv* by conjugation, using pRK2013 as helper plasmid in triparental matings (Figurski and Helinski, 1979). Plasmids used in this study are listed in Table S1.

### Plant material and inoculations

Plants were grown at day and night temperatures of 23° and 19°C, respectively, with 60/40% relative humidity and 16 h light. Plant lines used for the T3E screen are listed in Table S2. For detailed analysis of NHR of *N. tabacum* against *Xanthomonas*, the plant line *Nicotiana tabacum* L. cv. Petit Havana was used. Generation of the *Nbeds1* mutant *N. benthamiana* line was described previously (Ordon et al., 2016).

Two to four most expanded leaves of 5- to 9-week-old plants were used for inoculations. *Xcv* bacteria were hand-inoculated at an optical density (OD_600_) of 0.4 in 10 mM MgCl_2_ using a needleless syringe. For transient expression studies *in planta, A. tumefaciens* strains were resuspended in inoculation medium (10 mM MgCl_2_, 5 mM MES, pH 5.3, 150 μM acetosyringone) and hand-inoculated at OD_600_ = 0.8. For *in planta* growth curves, *Xcv* strains were inoculated at OD_600_ = 0.0004, and bacterial growth was determined as described (Bonas et al., 1991).

### Generation of expression constructs

For Golden Gate cloning, coding sequences of *xopC, xopG, xopO, xopP*, and *xopQ* were PCR-amplified from genomic DNA of *Xcv* 85-10 using oligonucleotides with *Bsa*I restriction sites (Table S3). Fragments were cloned into pUC57 or pJET1.2/blunt (Thermo Fisher Scientific), respectively, and then by *Bsa*I cut-ligation (Engler et al., 2008) into the expression vectors pBRM (Szczesny et al., 2010b) or pGGX1 for *Xcv*, and pGGA1 (Schulze et al., 2012), pGGA2 (Schreiber et al., 2015) and pGGA7, respectively, for *Agrobacterium*-mediated expression *in planta*. The binary vector pGGA7 contains the backbone of pBGWFS7 (Karimi et al., 2002), the chloramphenicol resistance-*ccd*B selection cassette from pGWB2 (Nakagawa et al., 2007), and allows *in planta* expression of genes 3′-translationally fused to 4 × c-Myc under the control of the cauliflower mosaic virus *35S* promoter. The *Xcv* expression vector pGGX1 contains the backbone of pBBR1MCS-5 (Kovach et al., 1995), the chloramphenicol resistance-*ccd*B selection cassette from pGWB2 (Nakagawa et al., 2007), and allows expression of genes 3′-translationally fused to a FLAG epitope under the control of the *lac* promoter. Cloning details are available upon request.

A DNA-fragment corresponding to the *NbEDS1a* cDNA and flanked by *Bpi*I restriction sites was synthesized as gBlocks fragment by Integrated DNA Technologies (IDT, Germany). The synthesized fragment did not contain internal *Bsa*I or *Bpi*I restriction sites, and codon usage was additionally altered to eliminate target sites of Cas9 nucleases used for generation of *eds1* mutant plants (Ordon et al., 2016). The fragment was cloned into pAGM1287 yielding pJOG285, and subsequently assembled together with pICH51277, pICH50010, and pICH41432 in pICH47732 to yield pJOG296 (Engler et al., 2014).

For Gateway cloning, coding sequences of *avrBsT, avrRxv, xopC*, and *xopH* were PCR-amplified from genomic DNA of *Xcv* 85-10 or *Xcv* 75-3 using oligonucleotides listed in Table S3. Fragments were cloned into pENTR/D-TOPO (Thermo Fisher Scientific) and subsequently recombined into the binary vectors pGWB5 (Nakagawa et al., 2007), pGWB6 (Nakagawa et al., 2007), or pK7FWG2 (Karimi et al., 2002) using Gateway® technology (Thermo Fisher Scientific).

### Construction of *xopQ* and *xopC* deletion strains

To generate *Xcv* 85-10Δ*xopQ*, 1-kb fragments upstream and downstream of *xopQ* were amplified from genomic DNA of *Xcv* 85-10 by PCR using oligonucleotides incorporating *Bsa*I restriction sites (Table S3). Because *xopC* is flanked by IS elements, *xopC* was only partial deleted. A 5′ fragment (298 bp upstream of *xopC* and the first 452 bp of *xopC*) and a 3′ fragment (last 327 bp of *xopC* and 121 bp downstream of *xopC*) were PCR-amplified from genomic DNA of *Xcv* 85-10 using oligonucleotides incorporating *Bsa*I restriction sites (Table S3). Corresponding 5′ and 3′ fragments were cloned into *Sma*I-digested pUC57 (Thermo Fisher Scientific) and subsequently into the suicide vector pOGG2 (Schulze et al., 2012). The resulting plasmids pOGG2:*xopC* and pOGG2:*xopQ* were conjugated into *Xcv* 85-10, and mutants were selected by PCR.

### Immunoblot analysis

For *Agrobacterium*-mediated expression studies, two 0.785 cm^2^ leaf discs per inoculated strain were ground in liquid nitrogen, resuspend in 130 μl 2 × Laemmli buffer and boiled. For analysis of protein synthesis in *Xcv*, bacteria were resuspended in 10 mM MgCl_2_ to OD_600_ = 0.4, 500 μl were pelleted, resuspended in 40 μl 2 × Laemmli and boiled. Proteins were separated by 10% SDS-PAGE and analyzed by immunoblotting. Strep Tag II Antibody HRP Conjugate (Merck Chemicals GmbH), anti-c-Myc (Roche Diagnostics) anti-GFP (Thermo Fisher Scientific) primary antibodies and horseradish peroxidase-labeled α-rabbit and α-mouse antibodies (GE Healthcare) were used.

## Results

### T3ES from *Xcv* induce necrosis or chlorosis on non-host *Solanaceae*

**Figure 1:**
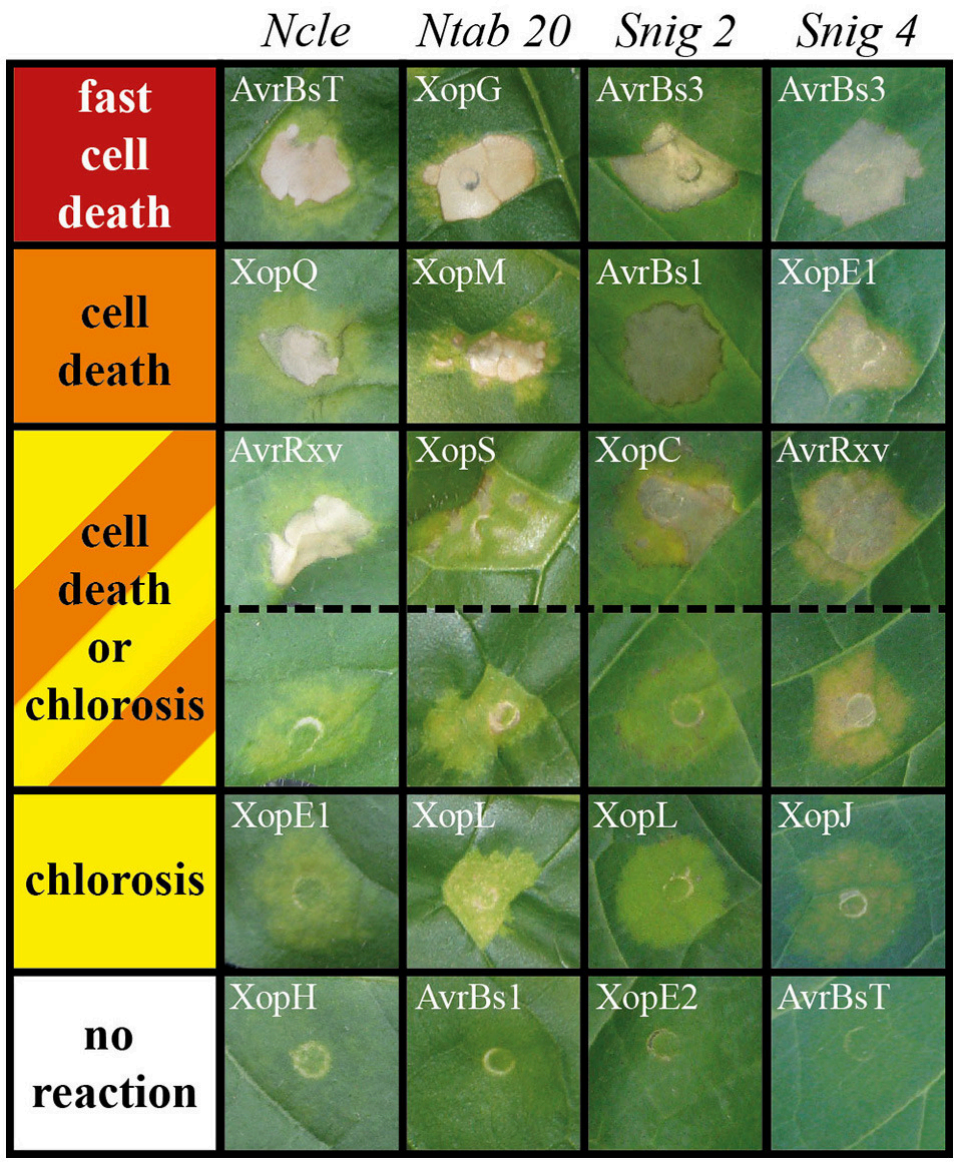
**Plant phenotypes resulting from *Agrobacterium*-mediated effector expression**. T3Es from *Xcv* were transiently expressed in 86 *Solanaceae* lines via *Agrobacterium*-mediated T-DNA transfer (see Table 2 for details). Plant reactions were classified into five groups, each represented by a color: red, fast cell death (3 dpi); orange, cell death (8 dpi); yellow, chlorosis (8 dpi); orange/yellow striped, chlorosis or cell death (8 dpi); white, no visible reaction (8 dpi). As examples, phenotypes of four plant lines after expression of different T3Es are shown. Plant lines were abbreviated according to Table S2. The *Xcv* effector causing the respective reaction is indicated. Photographs were taken 8 dpi.

To identify T3Es that induce a macroscopic reaction in non-host plants, 21 T3Es from different *Xcv* strains (Table 1) were synthesized via *Agrobacterium*-mediated transient expression in leaves of 86 non-host *Solanaceae* lines, mostly *Nicotiana* species (Table S2). Plant reactions were scored over 8 days and categorized into six classes as exemplified in Figure 1. Protein synthesis was probed by immunoblot analysis. Plant reactions and expression data are summarized in Tables 2, 3 and Table S4. Expression of GFP did not trigger visible reactions, indicating that *Agrobacterium* itself was not recognized by any plant line. Upon effector expression, plants showed a range of macroscopic responses, from no reaction to chlorosis and to more or less severe cell death. AvrBs2, AvrBsT, AvrRxv, XopE1, XopG, XopL, XopM, and XopQ caused reactions, often fast cell death, on the majority of the plant lines analyzed (Tables 2, 3). XopC, XopK, AvrBs3, XopJ, and XopV triggered reactions in a few lines tested, whereas only one plant line reacted to XopH (*Nnud*) and XopO (*Nvel*), respectively. Intriguingly, XopE2, XopI, and XopP never caused any visible reactions although they were mostly well expressed. We often observed no plant reaction in the infected tissue. Even in these cases, the majority of effectors was detectable by immunoblot, indicating that a lack of phenotype is not due to transformation efficiency.

**Table 1:** **T3Es from *Xcv* analyzed in this study**.

**Effector^a^**	**Comment(s)^b^**	**References**
AvrBs1	Unknown function	Ronald and Staskawicz, 1988; Escolar et al., 2001
AvrBs2	Putative glycerophosphoryl-diester phosphodiesterase	Kearney and Staskawicz, 1990; Zhao et al., 2011
AvrBs3	TAL effector family, transcriptional activator	Bonas et al., 1989; Kay et al., 2007
AvrBsT	YopJ/AvrRxv family, acetyltransferase	Escolar et al., 2001; Kim et al., 2010; Szczesny et al., 2010a; Cheong et al., 2014
AvrRxv	YopJ/AvrRxv family, putative cysteine protease and/or acetyltransferase	Whalen et al., 1993, 2008
XopB	HopD1 family, unknown function	Noël et al., 2001; Schulze et al., 2012
XopC	Putative haloacid dehalogenase-like hydrolase	Noël et al., 2003; Salomon et al., 2011
XopE1	HopX family, putative transglutaminase, N-myristoylation motif	Thieme et al., 2007
XopE2	HopX family, putative transglutaminase, N-myristoylation motif	Thieme et al., 2007
XopG	HopH family, putative zinc metalloprotease	Potnis et al., 2011; Schulze et al., 2012
XopH (AvrBs1.1)	Protein tyrosine phosphatase	Potnis et al., 2012
XopI	F-box motif	Schulze et al., 2012
XopJ	YopJ/AvrRxv family, putative cysteine protease and/or acetyltransferase	Noël et al., 2003; Üstün et al., 2013
XopK	Unknown function	Schulze et al., 2012
XopL	E3 ubiquitin ligase	Singer et al., 2013
XopM	Unknown function	Schulze et al., 2012
XopO	Homology to HopK1 and AvrRps4 (*P. syringae*)	Roden et al., 2004; Sohn et al., 2009
XopP	Unknown function	Roden et al., 2004
XopQ	HopQ1-1 family, putative inosine-uridine nucleoside N-ribohydrolase	Roden et al., 2004; Teper et al., 2014
XopS	Unknown function	Schulze et al., 2012
XopV	Unknown function	Schulze et al., 2012

a *T3Es isolated from Xcv strain 85-10 with the exception of AvrBs3 (from Xcv 82-8) and AvrBsT (from Xcv 75-3)*.

b *Putative molecular function, conserved motifs and/or homology to known T3Es from Pseudomonas and other Xanthomonas spp. For Pseudomonas effectors, the unified nomenclature was used (Lindeberg et al., 2005)*.

**Table 2:**
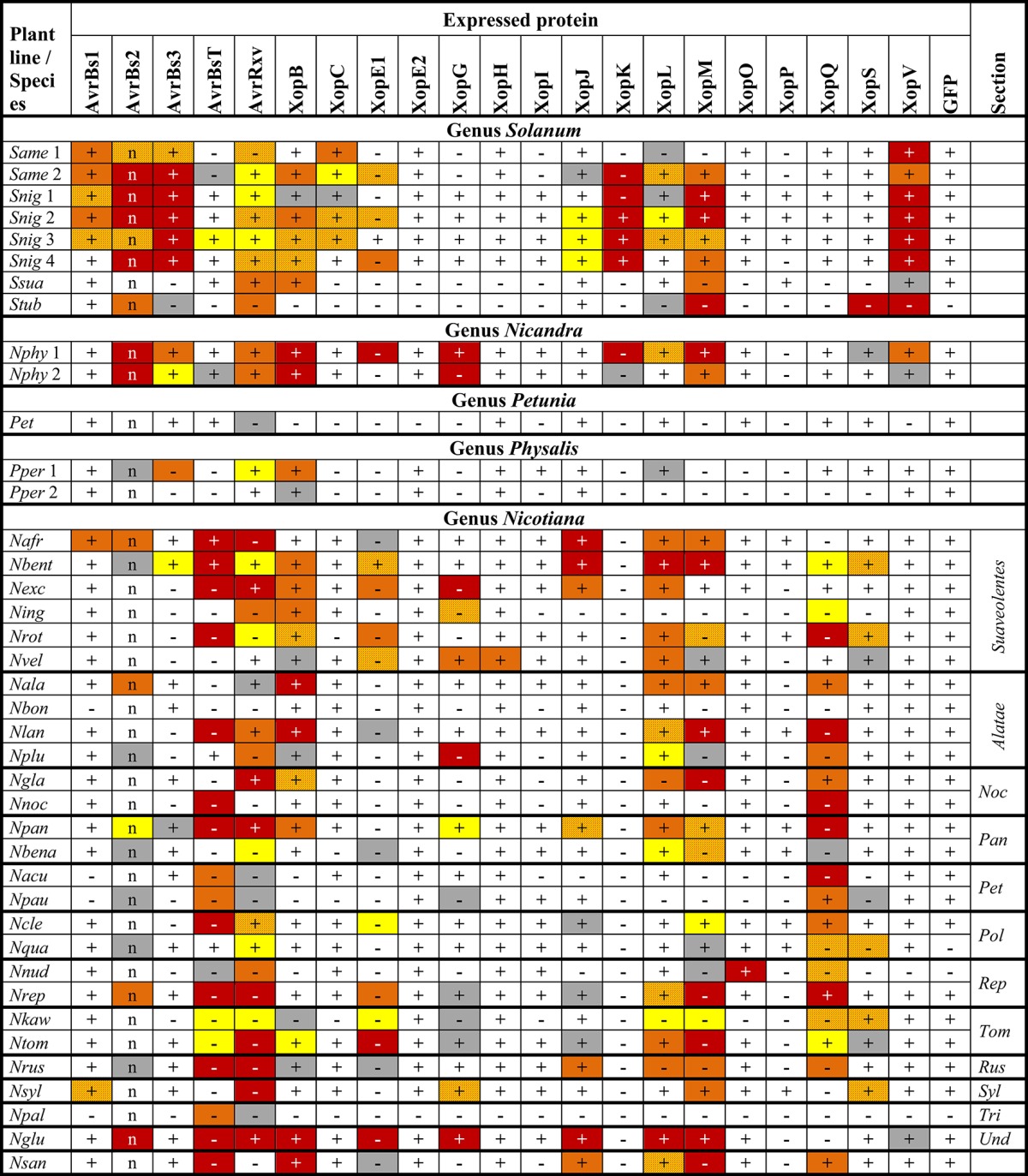
**Reactions of 40 solanaceous plants to *Agrobacterium*-mediated expression of *Xcv* T3Es**.

*Five plants per line, two leaves each, were inoculated with A. tumefaciens mediating the expression of the T3Es indicated or of GFP as control. Plant reactions were scored over 8 dpi and are represented by the color code as exemplified in Figure 1: red, strong necrosis on ≥7/10 spots (3 dpi); orange, weak necrosis on ≥7/10 spots (8 dpi); yellow, chlorosis on ≥7/10 spots (8 dpi); yellow/orange striped: chlorosis or cell death on ≥7/10 spots (8 dpi); white, no visible reaction on ≥7/10 spots (8 dpi); gray: data inconsistent (reactions on 4–6 spots). T3E expression analyzed by immunoblot is indicated: +, expression detectable; −, no expression detectable; n, expression not analyzed. Noc, Noctiflorae; Pan, Paniculatae; Pet, Petunioides; Pol, Polydicliae; Rep, Repandae; Tom, Tomentosae; Rus, Rusticae; Syl, Sylvestres; Tri, Trigonophyllae; Und, Undulatae*.

**Table 3:**
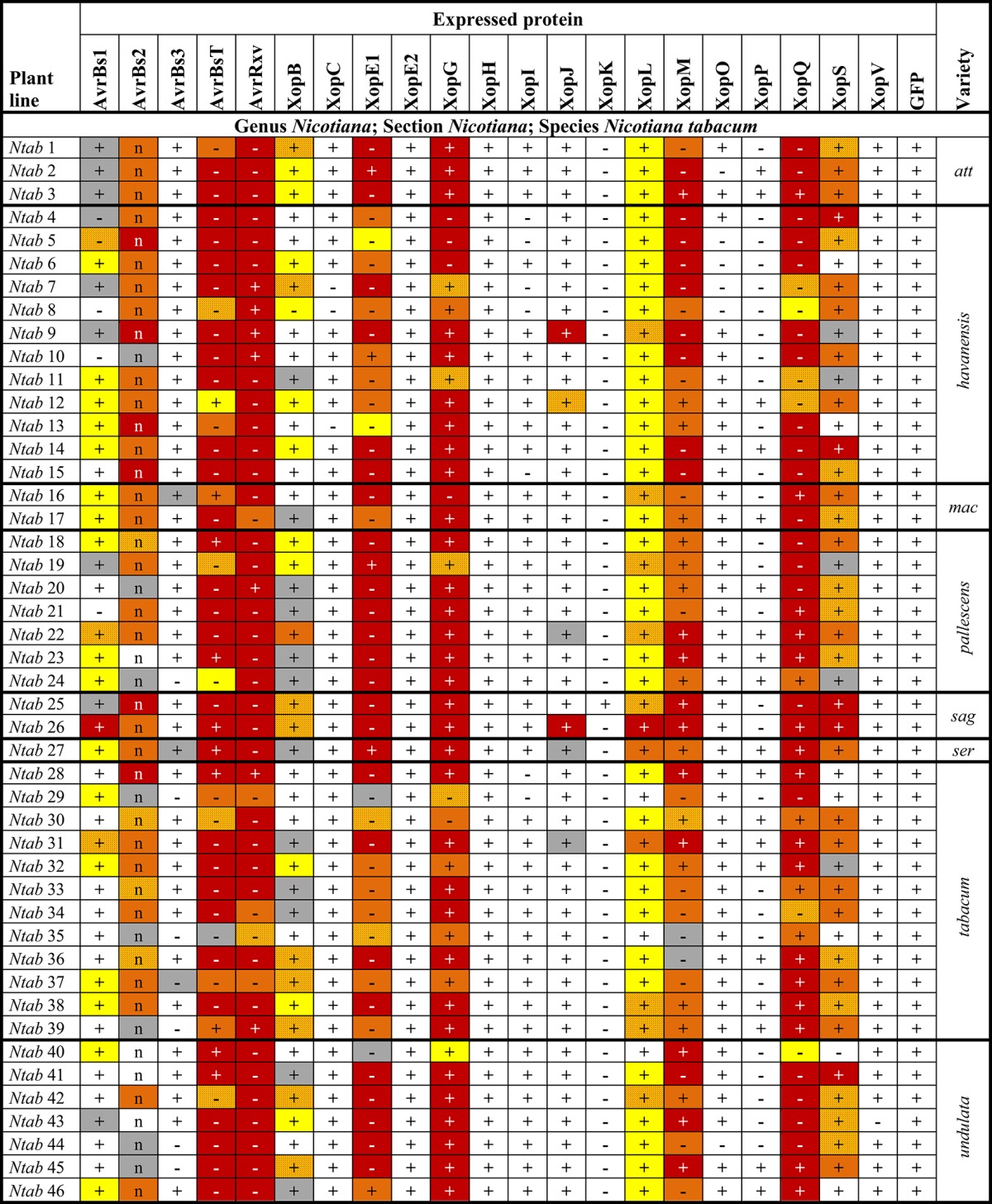
**Reactions of 46 *Nicotiana tabacum* plant lines to *Agrobacterium*-mediated expression of *Xcv* T3Es**.

*The same experimental procedure and color code as described in Table 2 was used. att, attenuata; mac, macrophylla; sag, sagittata; ser, serotina*.

After the first survey, a subset of 18 plant accessions encompassing most phylogenetic groups was tested again in at least two additional independent experiments which generally confirmed the first results (Table S5).

### Members of T3E families trigger diverse plant-reaction patterns

**Figure 2:**
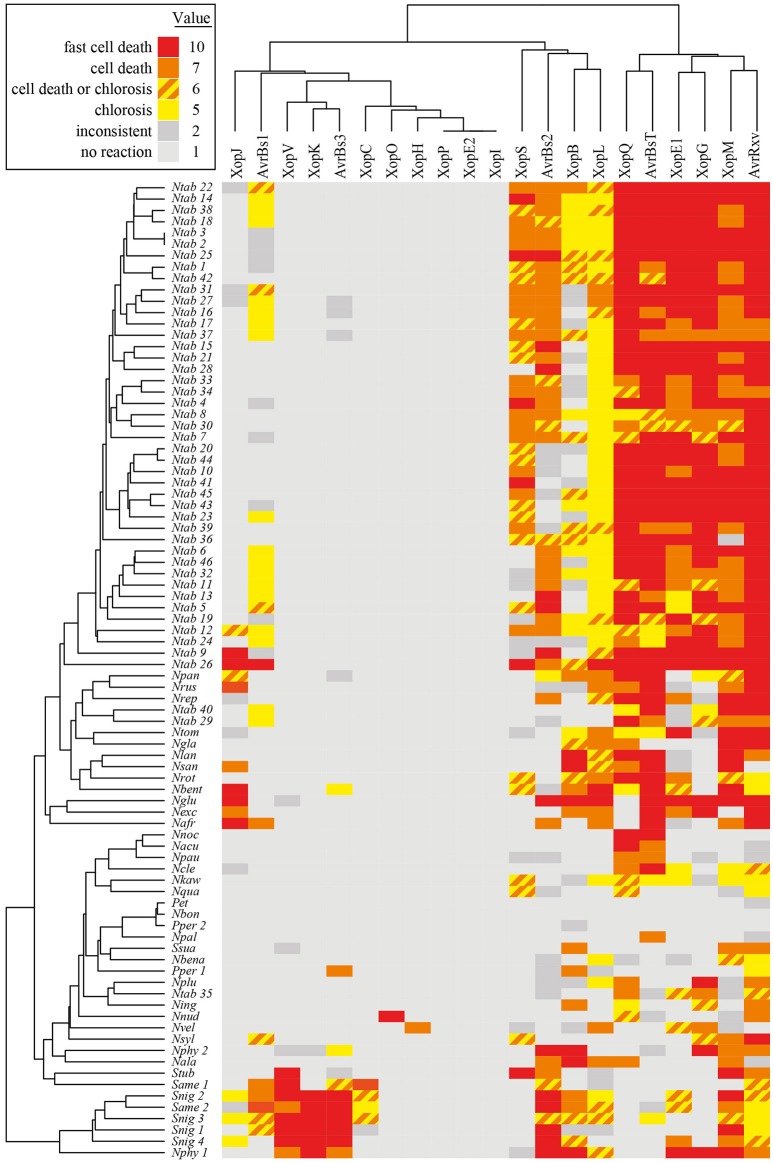
**Plant reactions to *Agrobacterium*-mediated transient expression of *Xcv* T3Es**. Heatmap representation of effector responses in 86 different non-host *Solanaceae* plant lines (for abbreviations see Table S2). Five plants per line, two leaves per plant, resulting in 10 spots per *Agrobacterium* strain, were inoculated with *Agrobacterium* strains mediating expression of the T3Es indicated on top. Plant reactions observed on at least 7/10 spots were classified as follows: fast cell death (3 dpi); cell death (6 dpi); chlorosis (6 dpi); chlorosis or cell death (6 dpi); no visible reaction (6 dpi). Reactions on only 4-6/10 spots were judged to be inconsistent. Plant reactions were visualized in a heatmap using the color code indicated. Each reaction type was assigned a value serving as the basis for clustering. The dendrogram shows the results of hierarchical clustering using average linkage and euklidean distance measures for T3Es and plant genotypes, respectively.

Hierarchical cluster analysis was performed to identify potential commonalities between T3Es with respect to the induced plant reactions. Since we aimed at the identification of T3Es that are recognized in solanaceous non-host plants, special emphasis was laid on fast cell death, i.e., HR-like reactions, by scoring of the observed reactions on a numerical scale from 1 (no reaction) to 10 (fast cell death). Hierarchical cluster analysis of effectors and plant accessions revealed two branches of T3Es (Figure 2): T3Es, which triggered reactions on most lines of the genus *Nicotiana* (AvrBsT, AvrRxv, XopE1, XopG, XopM, XopQ) and T3Es, which induced reactions less frequently (all other T3Es). Only a few T3Es showed similar reaction patterns: AvrBs3, XopK, and XopV, which induced cell death in most *Solanum* species cluster together, as well as T3Es that triggered visible reactions in only few lines (XopC, XopE2, XopH, XopI, XopO, and XopP). All other T3Es triggered rather unique reaction patterns (Figure 2). Considering the overrepresentation of *N. tabacum* lines (Table S2), one line of each *N. tabacum* variety was randomly selected and hierarchical cluster analysis repeated (Figure S1). This led to only minor changes in T3E clustering (compare Figure 2 and Figure S1). The tested T3E set contained three members of the YopJ/AvrRxv T3E family (AvrBsT, AvrRxv, and XopJ) and two members of the HopX T3E family (XopE1 and XopE2). Interestingly, members of a given T3E family did not group together in hierarchical cluster analysis. Thus, the classification into a “family” does not allow conclusions or the predictions about a T3E's capacity to induce plant reactions or about their putative recognition via corresponding *R* genes/R proteins.

### Conservation of putative *R* genes

The observed T3E-induced plant reactions in different species might rely on the presence of corresponding *R* genes. Among our set of plant lines, in particular the *Nicotiana* phylogeny has been extensively studied. Based on phylogenetic studies, e.g., sequence analyses of plastid- and nuclear-encoded genes and genomic *in situ* hybridization experiments (Chase et al., 2003; Clarkson et al., 2010; Kelly et al., 2013), the genus *Nicotiana* has been divided into 13 sections (Knapp et al., 2004). To study conservation of putative *R* genes in this genus in more detail, representative lines of all sections were tested. No T3E triggered a reaction in all tested *Nicotiana* lines (Table 2 and Table S2). We furthermore included six species of section *Suaveolentes* and four species of section *Alatae* to test for conservation of putative *R* genes within a given section. Since none of the tested T3Es triggered reactions in all representatives of the two sections (Table 2), putative corresponding *R* genes within *Suaveolentes* and *Alatae* appear not to be conserved.

Finally, 46 members of the species *N. tabacum* (sect. *Nicotiana*) were analyzed. AvrBsT, AvrRxv, XopE1, XopG, XopL, XopM, and XopQ triggered consistent reactions in all or most lines of the species *N. tabacum* (at least 43 out of 46 lines), suggesting a high conservation of putative corresponding *R* genes (Table 3). Four T3Es triggered consistent reactions in 21–34 *N. tabacum* lines tested: AvrBs1 (21/46), AvrBs2 (34/46), XopB (21/46), and XopS (34/46). Putative *R* genes recognizing these T3Es appear less conserved, but retain a high persistence among *N. tabacum* lines.

Taken together, some putative *R* genes are conserved within the species *N. tabacum*, whereas no conservation was observed within the superordinate phylogenetic units section and genus.

### XopQ is a host range determinant in a number of *Nicotiana* species

**Figure 3:**
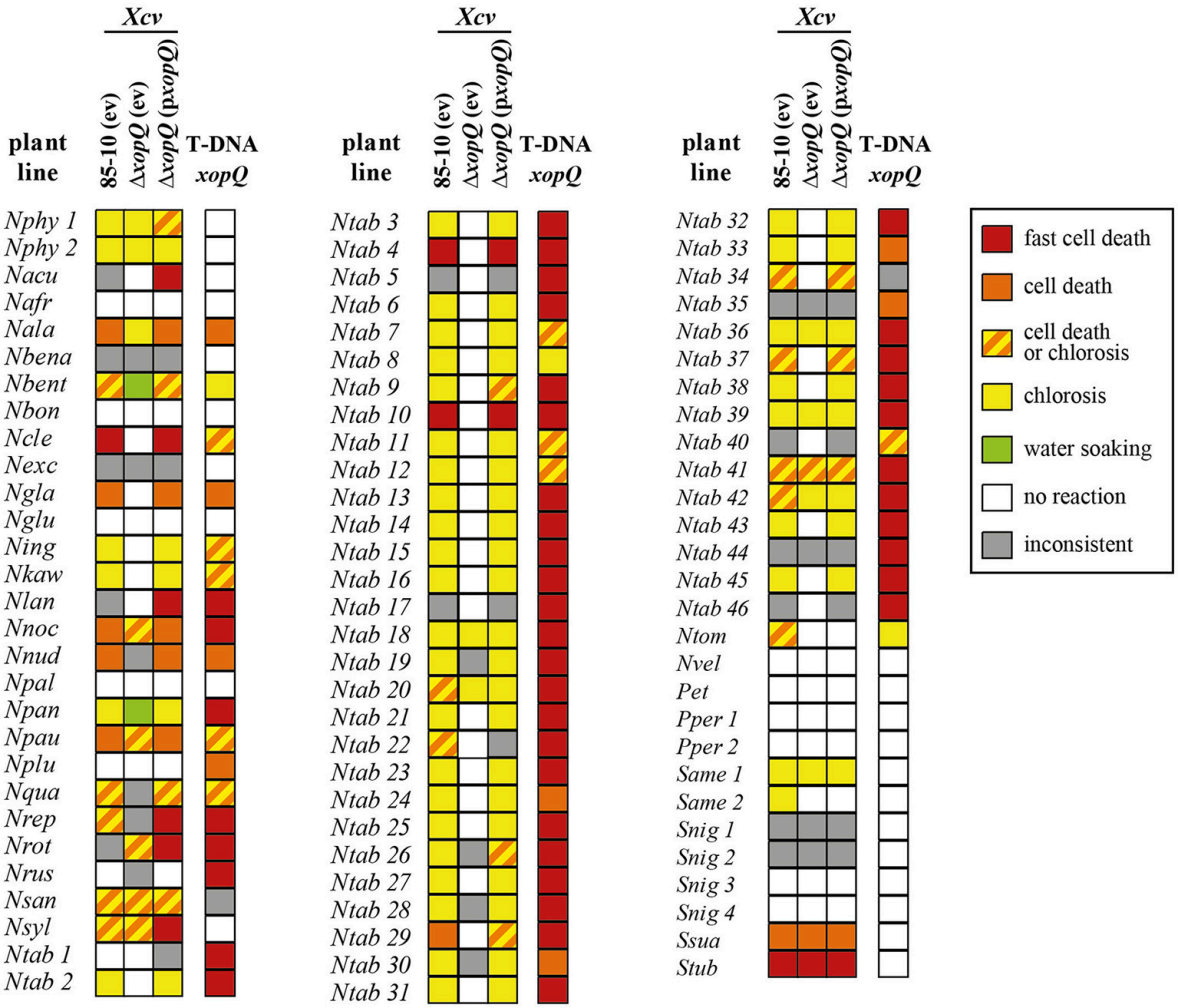
**Avirulence activity of XopQ is restricted to *Nicotiana* species**. Eighty-six different non-host *Solanaceae* plant lines (for abbreviations see Table S2) were inoculated with *Xcv* strains 85-10 and *Xcv* 85-10Δ*xopQ*, harboring empty vector (ev) or pBRM:xopQ (p*xopQ*), at OD_600_ = 0.4; reactions were scored for 6 days. Five plants per line with two leaves per plant were inoculated resulting in 10 spots per analyzed *Xcv* strain. Plant reactions are indicated according to the following color code: red, fast cell death (3 dpi); orange, cell death (6 dpi); yellow, chlorosis (6 dpi); orange/yellow striped, chlorosis or cell death (6 dpi); green, water-soaked lesions (6 dpi); white, no visible reaction (6 dpi). Colors were assigned if the same type of reaction was observed on ≥7/10 spots, reactions on only 4-6/10 spots were judged inconsistent, indicated in gray. Plant phenotypes 8 dpi of *Agrobacterium* mediating *xopQ* expression are indicated on the right-hand side of each column.

**Figure 4:**
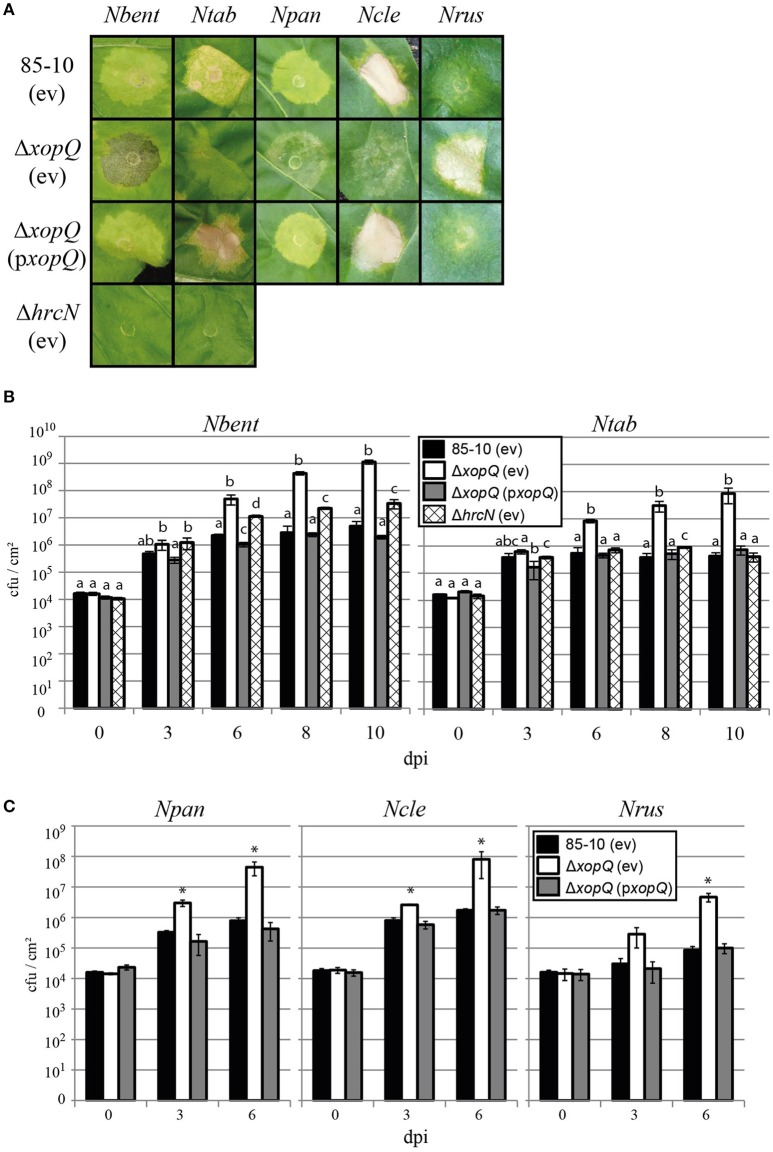
**XopQ shows avirulence activity in *Nicotiana* spp**. Five non-host lines were infected with *Xcv*: *N*. *benthamiana* (*Nbent*), *N. tabacum* (*Ntab*), *N. paniculata* (*Npan*), *N. clevelandii* (*Ncle*), and *N. rustica* (*Nrus*). **(A)** Leaves were inoculated with *Xcv* strains 85-10, 85-10Δ*xopQ*, and 85-10Δ*hrcN*, harboring empty vector (ev) or pBRM:xopQ (p*xopQ*) at OD_600_ = 0.4. Photographs were taken 6 dpi (*Nbent, Ntab*), 7 dpi (*Npan, Ncle*) and 12 dpi (*Nrus*), respectively. **(B,C)** Bacterial growth of *Xcv* strains in leaves was tested. The same *Xcv* strains as above were inoculated and bacterial multiplication was monitored over a period of 10 days. Values represent the mean of three samples from three different plants. Error bars indicate standard deviation. Different letters represent statistically significant differences; asterisks indicate statistically significant differences when compared to the wild-type strain (two sided *t*-test, *P* < 0.05). Experiments were repeated at least twice with similar results.

Strikingly, XopQ expression induced necrotic or chlorotic reactions exclusively in *Nicotiana* species (Figure 2, Table 2), suggesting the presence of a XopQ-specific *R* gene (*R*_*xopQ*_) in most members of this genus. We speculated that XopQ is also recognized during infection of *Nicotiana* spp. with *Xcv* and therefore contributes to *Xcv*-induced NHR. To test the influence of XopQ on NHR, all 86 *Solanaceae* lines were infected with the wild-type strain *Xcv* 85-10, the *Xcv* 85-10Δ*xopQ* deletion mutant and an *Xcv* 85-10Δ*xopQ* strain ectopically expressing *xopQ*. *Xcv* 85-10Δ*xopQ* caused weaker or no reactions compared to the wild-type strain on approximately two-thirds of the accessions tested (Figure 3). The plant phenotypes after *Xcv* infection correlated well with reactions observed after *Agrobacterium*-mediated XopQ expression: If T-DNA delivery of *xopQ* induced a cell death or chlorosis, *Xcv*-induced reactions also were *xopQ*-dependent (Figure 3). Intriguingly, two plant lines, *N. benthamiana* (*Nbent*) and *N. paniculata* (*Npan*), showed water-soaked lesions after infection with *Xcv* 85-10Δ*xopQ*, whereas infection with the wild-type and the complemented Δ*xopQ* mutant triggered chlorotic or cell death reactions (Figures 3, 4A).

Similarly to the transient expression via *Agrobacterium*, a subset of 18 plant accessions encompassing most phylogenetic groups was analyzed in at least two additional independent experiments. Results largely confirmed the reactions shown in Figure 3 (Table S5). Inoculation of *Xcv* 85-10Δ*hrcN* (Lorenz and Büttner, 2009), a T3SS-deficient and non-pathogenic mutant, never resulted in visible reactions (Table S5). Thus, macroscopic NHR reactions depend on T3E translocation, whereas T3SS-independent recognition of *Xcv*, i.e., during PTI, failed to induce visible NHR reactions.

Next, we determined whether XopQ contributes to bacterial multiplication in leaves of *N. benthamiana* (*Nbent*), *N. tabacum* (*Ntab*), *N. paniculata* (*Npan*), *N. clevelandii* (*Ncle*), and *N. rustica* (*Nrus*). In these lines, *xopQ* differentially determines the *Xcv*-induced NHR reaction: *Xcv* 85-10 induces a *xopQ*-dependent chlorotic reaction in *Nbent, Ntab*, and *Npan* and a HR-like reaction in *Ncle* (Figure 4A). *Xcv* 85-10Δ*xopQ* triggered water soaking on *Nbent* and *Npan* and nearly no visible reactions on *Ntab* and *Ncle*. *Nrus* was the only plant line in which *Xcv* 85-10Δ*xopQ* triggered cell death, whereas *Xcv* 85-10 caused no visible reactions (Figures 3, 4A). As shown in Figures 4B,C, *Xcv* 85-10 moderately multiplied in all plant lines, whereas *Xcv* 85-10Δ*xopQ* grew significantly better. We also analyzed *in planta* growth of the T3S-deficient strain *Xcv* 85-10Δ*hrcN* in *Ntab* and *Nbent*. Interestingly, *Xcv* 85-10Δ*hrcN* multiplied significantly better in *Nbent* than *Xcv* 85-10 (Figure 4B) indicating a strong impact of ETI on NHR of *Nbent*. Taken together, in all *Nicotiana* species analyzed, XopQ displays an avirulence activity triggering plant defenses and restricting the growth of *Xcv* in the leaf tissue.

### XopC determines the *Xcv*-induced phenotype in *S. americanum*

**Figure 5:**
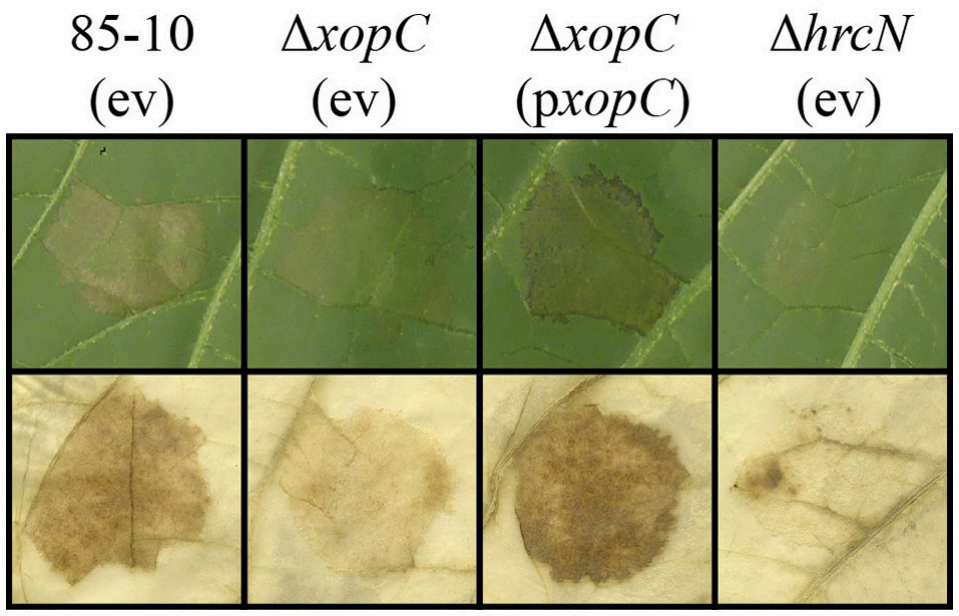
**XopC influences *Xcv*-mediated non-host resistance in *Solanum americanum***. *Solanum americanum* (*Same 1*) leaves were inoculated with *Xcv* strains 85-10, 85-10Δ*xopC*, and 85-10Δ*hrcN*, harboring an empty vector (ev) or pBRM:*xopC* (p*xopC*), at OD_600_ = 0.4. Plant reactions were documented 5 dpi (upper panel) and 3 dpi (lower panel). For better visualization of cell death reactions at 3 dpi, the leaf was bleached in EtOH (lower panel). The experiment was repeated twice with similar results.

As described above, XopQ affected *Xcv*-mediated NHR reactions in all *Nicotiana* plant lines in which *Agrobacterium*-mediated expression of XopQ triggered a reaction. We wondered if this is also true for other T3Es. In contrast to XopQ, transient expression of XopC exclusively induced plant reactions in lines of the genus *Solanum* (Table 2). We speculated that XopC contributes to *Xcv*-induced reactions in these plant lines and generated a *xopC* deletion mutant. As shown in Figure 5, *Xcv* 85-10Δ*xopC* induced weaker reactions than *Xcv* 85-10 in *S. americanum* (*Same 1*), which could be complemented by ectopic expression of *xopC*. Deletion of *xopC* did not affect visible reactions in *N. benthamiana* and *N. tabacum* to infection with *Xcv* (data not shown). Thus, similarly to recognition of XopQ, also XopC contributes to *Xcv*-induced phenotypes in certain non-host plants.

### XopQ-mediated recognition in *N. benthamiana* depends on *EDS1*

**Figure 6:**
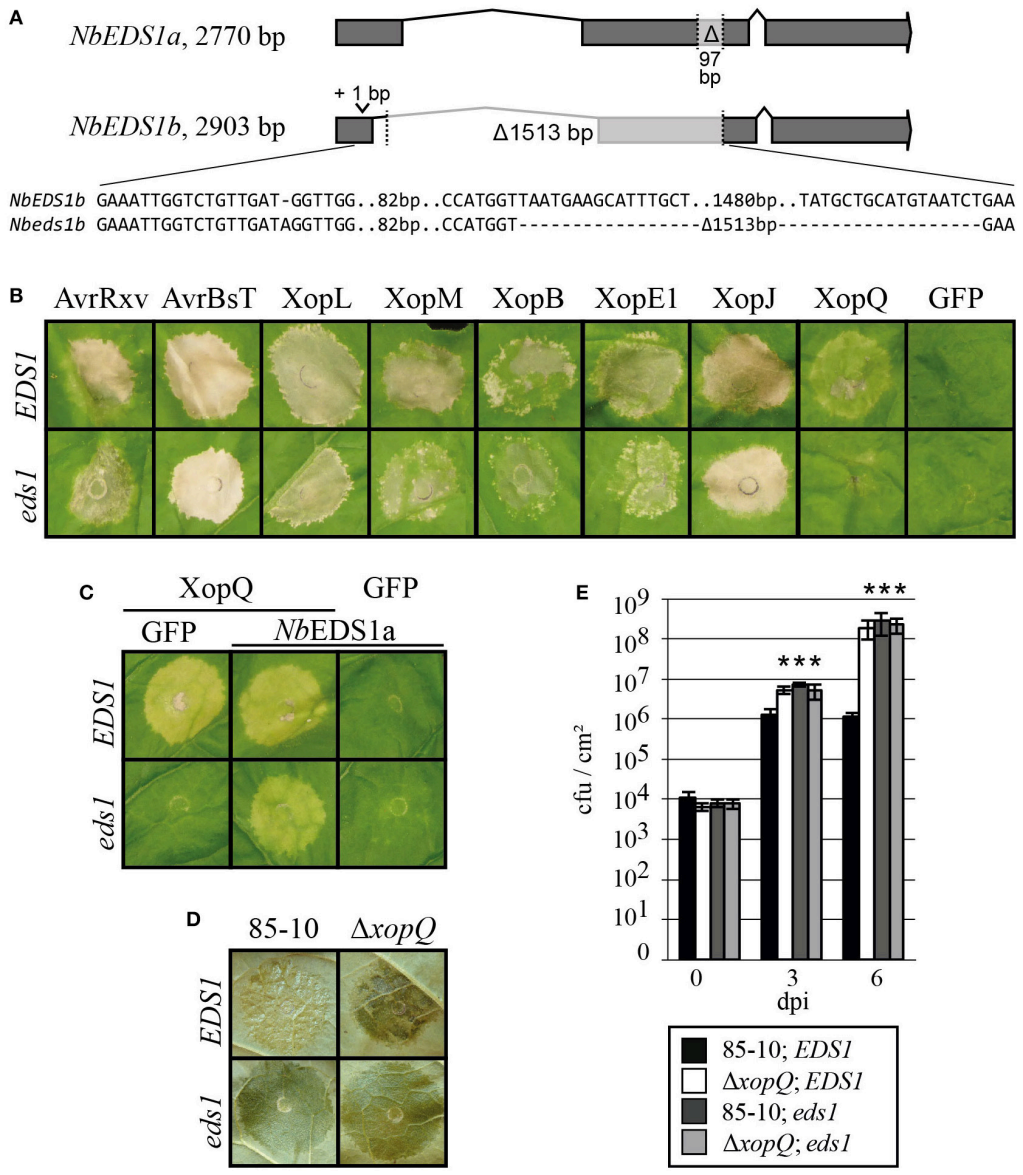
**Recognition of XopQ and *Xcv* in *N. benthamiana* depends on *EDS1*. (A)** Schematic representation of *EDS1* loci in *N. benthamiana*. The genomic mutations harbored in the *Nbeds1a-1* line are indicated. **(B–E)** Leaves of wild-type *N. benthamiana* (*EDS1*) and *Nbeds1a-1* (*eds1*) mutant plants were inoculated. **(B)** *A. tumefaciens* strains mediating expression of the indicated T3Es and GFP with OD_600_ = 0.8 were inoculated. Photographs were taken 7 dpi. **(C)** *A. tumefaciens* strains mediating expression of XopQ, GFP or *Nb*EDS1a with OD_600_ = 0.8 were mixed in a 1:1 ratio and inoculated. Photographs were taken 10 dpi. **(D)** Inoculation of *Xcv* 85-10 and 85-10Δ*xopQ* at OD_600_ = 0.4. Phenotypes were documented 7 dpi. **(E)** Bacterial multiplication was monitored over a period of 6 days after inoculation of *Xcv* 85-10 and 85-10Δ*xopQ* at OD_600_ = 0.0004. Values represent the mean of three samples from three different plants. Error bars indicate standard deviations. Asterisks indicate significant differences compared to *Xcv* 85-10 in *EDS1* plants (two-sided *t*-test, *P* < 0.05). Experiments were repeated at least twice with similar results.

In most cases, T3E recognition takes place within the plant cell via corresponding NLR-type R proteins (Khan et al., 2016). TIR domain-containing TNLs represent one large NLR subgroup, and TNL-mediated immunity required the lipase-like protein EDS1 in *N. benthamiana* (Peart et al., 2002), tomato (Hu et al., 2005) and *Arabidopsis thaliana* (Aarts et al., 1998; Wirthmueller et al., 2007). We employed a recently reported *Nbeds1a-1* line to test EDS1 dependency of T3E-induced plant reactions in *N. benthamiana*, which encodes two *EDS1* orthologs, *NbEDS1a* and *NbEDS1b* (Ordon et al., 2016). *Nbeds1a-1* was reported to contain a 97-bp deletion in exon 2 of *NbEDS1a*, which was generated using Cas9-based nucleases. Since employed guide RNAs also targeted *NbEDS1b*, this locus was sequence-verified. Indeed, the *Nbeds1a-1* line additionally contained both a point mutation and a large deletion at the *NbEDS1b* locus (Figure 6A). However, this line will be further referred to as *Nbeds1a-1*, since *NbEDS1b* is most likely a pseudogene (Figure S2). When T3Es were transiently expressed in *Nbeds1a-1* leaf tissues, plant reactions induced by AvrBsT, AvrRxv, XopE1, XopJ, XopL, and XopM were unaltered, indicating EDS1-independent recognition of these effectors (Figure 6B). In contrast, XopQ-induced chlorosis was abolished on *eds1* mutant plants, suggesting activation of an EDS1-dependent resistance pathway (Figure 6B). A transient complementation assay was used to unequivocally show EDS1-dependent recognition of XopQ in *N. benthamiana*. XopQ or GFP were transiently co-expressed with *Nb*EDS1a in wild-type or *eds1* mutant leaf tissues (Figure 6C). XopQ expression induced chlorosis on wild type, but not *eds1* plants, and chlorosis was restored upon co-expression of *Nb*EDS1b (Figure 6C). Thus, the putative *R*_*xopQ*_ for recognition of XopQ most likely encodes a TIR-type NLR protein.

To analyze the role of *EDS1* in the NHR of *N. benthamiana* against *Xcv*, we inoculated *Xcv* 85-10 and *Xcv* 85-10Δ*xopQ* into *N. benthamiana* wild-type (*EDS1*) and *Nbeds1a-1* (*eds1*) plants. *Xcv* 85-10 triggered no disease symptoms and showed a moderate growth, whereas *Xcv* 85-10Δ*xopQ* multiplied significantly better and caused disease symptoms in wild-type *N. benthamiana* (Figures 6D,E). In *eds1* plants, both *Xcv* strains caused disease and multiplied equally well (Figures 6D,E). Thus, EDS1 is essential for the NHR of *N. benthamiana* against *Xcv* 85-10, most likely due to its essential role in XopQ recognition via a corresponding TIR-type NLR.

## Discussion

### Different *Solanaceae* encode a diverse set of putative *R* genes

Our work is the first larger study on reactions caused by *Xanthomonas* T3Es in non-host plants. Plant phenotypes upon T3E expression reached from fast, HR-like cell death over chlorotic reactions to no visible reaction. T3E-induced cell death reactions are a hallmark of ETI (Henry et al., 2013) and, therefore, suggest the presence of one or several corresponding *R* genes/R proteins. Chlorotic reactions might also result from ETI, as shown for recognition of the *Pseudomonas syringae* T3E AvrB by the TNL TAO1 in *A. thaliana* (Eitas et al., 2008). In some cases, however, the observed phenotypes might result from a virulence-associated activity of the respective strongly expressed effector and occur independently of an *R* gene/R protein. Transient expression of T3Es in different *Solanaceae* species led to diverse reaction patterns (Tables 2, 3), suggesting variable sets of putative *R* genes among *Solanaceae* or different sensitivities of plant lines to virulence activities of T3Es. A genetic variation of *R* genes has often been described, whereas a genetic variation of plant susceptibility against T3Es virulence activities is rarely reported. Therefore, we basically interpret our data according to the presence or absence of putative *R* genes. However, this simplification requires further analysis, i.e., the isolation of corresponding *R* genes. The number of plant *R* genes varies strongly in different *Solanaceae* species, e.g., 2042 NLRs were annotated in pepper (Chiltepin), whereas tomato (Heinz1706) only encodes 478 NLRs (Wei et al., 2016). Furthermore, a high evolution rate of *R* genes and *R* gene clusters was shown, e.g., in various *Solanaceae* plants (Jupe et al., 2012; Quirin et al., 2012; Andolfo et al., 2013), *Fabaceae* (Zheng et al., 2016), *Arabidopsis lyrata* (Buckley et al., 2016) and grasses (Yang et al., 2008, 2013; Luo et al., 2012; Zhang et al., 2014). We observed variable plant responses between members of the section *Nicotiana* and even between closely related members of the species *N. tabacum*, indicating dynamic acquisition and loss of *R* genes.

### Conservation of putative *R* genes in *N. tabacum* lines

**Figure 7:**
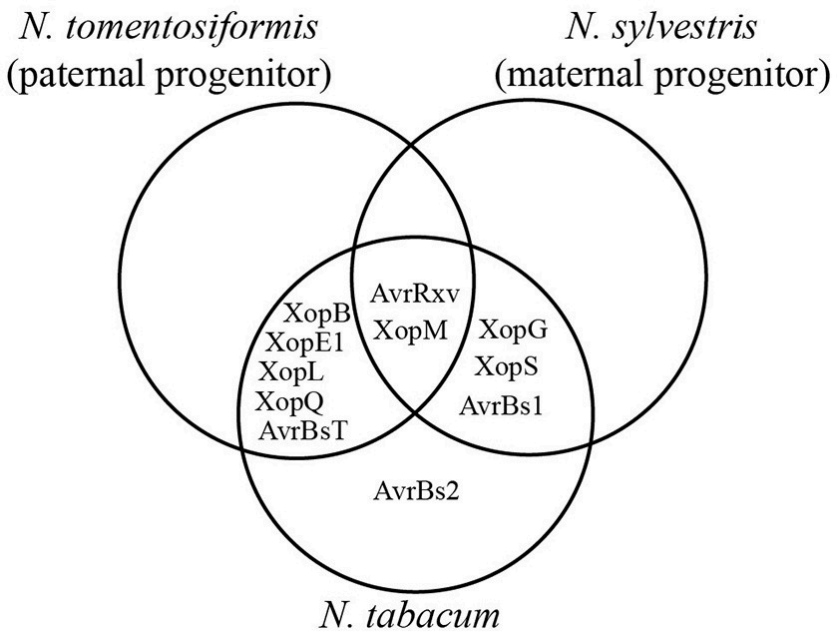
***N*. *tabacum* recognizes T3Es similarly to its progenitors**. T3Es which trigger consistent plant reactions in *N. tomentiformis, N. sylvestris* and at least 21 of 46 tested *N. tabacum* lines were compared. For details see Table 3.

*N. tabacum* is an allotetraploid species which originated approximately 200,000 years ago from an interspecific cross of *N. sylvestris* (2n = 24, maternal progenitor) with *N. tomentosiformis* (2n = 24, paternal progenitor) (Leitch et al., 2008; Sierro et al., 2013, 2014). Interestingly, two sets of T3Es triggered consistent reactions in *N. tomentosiformis* (AvrBsT, AvrRxv, XopB, XopE1, XopL, XopM, and XopQ) and *N. sylvestris* (AvrBs1, AvrRxv, XopG, XopM, and XopS), respectively, with AvrRxv and XopM being recognized in both species. Taken together, one can speculate that *R* genes recognizing these 10 T3Es were combined upon genome fusion in *N. tabacum* (Figure 7). Indeed, putative *R* genes recognizing seven of the 10 T3Es (AvrBsT, AvrRxv, XopE1, XopG, XopL, XopM, and XopQ) appear to be conserved in *N. tabacum* until today. By contrast, putative *R* genes recognizing the T3Es AvrBs1, XopB and XopS got (functionally) lost in a number of cultivated *N. tabacum* lines, e.g., *Ntab* 8, 36, 39 (no AvrBs1-mediated reaction) and *Ntab* 5, 16 (no XopB-mediated reaction). Interestingly, AvrBs2 also triggered reactions in most *N. tabacum* lines, but not in lines of the progenitor species *N. tomentosiformis* or *N. sylvestris* tested here, suggesting loss of the putative corresponding *R* genes in *N. tomentosiformis* and *N. sylvestris* or gain in *N. tabacum* in the course of evolution (Table 3, Figure 7). The T3E AvrBs2 is recognized in pepper ECW-20R plants (Minsavage et al., 1990) and is a virulence factor across xanthomonads (Kearney and Staskawicz, 1990).

Out of the 46 tested *N. tabacum* lines, only *Ntab* 9, *Ntab* 12 and *Ntab* 26 showed consistent reactions to XopJ (Table 3). A plausible explanation could be that these lines acquired XopJ-specific *R* genes only recently. The same might be true for *N. velutina* (*Nvel*) and *N. nudicaulis* (*Nnud*), which were the only lines recognizing XopH and XopO, respectively (Table 2). As mentioned above, the observed reaction patterns might also rely on a genetically determined variation of plant susceptibility against the virulence activity of a given T3E.

### ETI contributes to the *Xcv*-induced NHR

Up to now it was largely unknown whether *Xcv* translocates T3Es into non-host plants and whether ETI is induced during NHR. We identified XopQ as avirulence determinant within several non-host plant lines and found that XopC contributes to *Xcv*-induced plant reactions during infection of *S. americanum*. These results indicate that *Xcv* translocates T3Es into the plant cells of non-host species. In contrast to *Xcv* 85-10, the T3SS-deficient strain *Xcv* 85-10Δ*hrcN* did not induce phenotypic reactions on non-host plants (Table S5). We, therefore, assume that ETI significantly contributes to NHR against *Xcv*. Similarly, ETI also contributes to the NHR of diverse plant lines during interaction with *Pseudomonas syringae* (Lindeberg et al., 2009, 2012; Senthil-Kumar and Mysore, 2013).

Since *Xcv* 85-10Δ*hrcN* multiplied significantly better than *Xcv* 85-10 in *N. benthamiana* (Figure 4B), PTI appears to restrict *Xcv* growth in *N. benthamiana* less efficiently than the combination of PTI and ETI. This is reminiscent of a recent model by Cui et al. (2015) which describes PTI as a balance of positive and negative immunity signals to prevent plants from overreactions to harmless microbes. Initiation of ETI, signaling the presence of a serious pathogen threat, dampens negative regulation of PTI, resulting in an efficient plant immunity to halt the infection (Cui et al., 2015). This, however, cannot be generalized as in *N. tabacum* the *hrcN* deletion strain affected NHR phenotypes but not *in planta* growth of *Xcv*. Future studies on the interaction of *Xcv* with *N. benthamiana* and *N. tabacum* might help to understand quantitative differences in plant immunity responses.

### XopQ is probably recognized by a TIR-type NLR in *Nicotiana* spp.

Here, we identified XopQ as a key host range factor in *Xcv* for the interaction with *Nicotiana* species. A recent study performed at the same time as ours also identified XopQ as a host range factor in *N. benthamiana* and proposed a XopQ-specific R protein, R_XopQ_ (Schwartz et al., 2015). In most cases, *Xcv* 85-10Δ*xopQ* induced weaker NHR reactions on *Nicotiana* spp. compared to *Xcv* 85-10 and only caused disease on *N. benthamiana* and *N. paniculata*. This might be due to the recognition of at least one additional T3E or due to the inability to modulate virulence targets in most non-host plants.

The finding that *N. benthamiana EDS1* is essential for the XopQ-mediated NHR suggests that *R*_*xopQ*_ encodes a TIR-type NLR. To our knowledge, this is the first report on the role of *EDS1* in NHR against a bacterial pathogen in *N. benthamiana*. It is worth to note that several *Xcv* T3Es (Figure 6B) can induce HR-like reactions when expressed in *N. benthamiana*, but the deletion of *xopQ* in *Xcv* 85-10 is sufficient to abolish NHR, and allows full plant colonization and disease symptom formation (Figure 6E; Schwartz et al., 2015). Thus, remaining T3Es are either translocated at low levels, below a threshold for avirulence activity, or avirulence activities might be suppressed by simultaneously translocated other T3Es. XopQ was identified as the only effector recognized in an *EDS1*-dependent manner, and *Xcv* 85-10 and *Xcv* 85-10Δ*xopQ* strains grew equally well on *eds1* mutant plants. These observations suggest that XopQ is most likely the only *Xcv* T3E recognized in an *EDS1*-dependent manner in *N. benthamiana*, and resistance defects in *eds1* mutant lines do not extend beyond abolished TNL signaling.

Interestingly, there are several parallels between recognition of XopQ from *Xcv* and recognition of the XopQ homolog from *Pseudomonas syringae*, HopQ1. As XopQ, HopQ1 from *P. syringae* DC3000 induces chlorosis in *N. benthamiana* (Wroblewski et al., 2009) and a fast cell death in *N. tabacum* (Li et al., 2013a) after transient expression. Additionally, HopQ1 restricts host range of *P. syringae* strains in *N. benthamiana* dependent on SGT1, indicating the presence of a HopQ1-specific R protein (Wei et al., 2007; Ferrante et al., 2009). In case of HopQ1, its virulence activity can be clearly separated from its avirulence activity because the nucleoside hydrolase-like domain of HopQ1 and the interaction of HopQ1 with host 14-3-3 proteins contribute to virulence but are dispensable for recognition in *N. tabacum* (Li et al., 2013a,b). It could very well be that recognition of XopQ and HopQ1 is mediated by a single TIR-type NLR. Identification of the representative *R* gene might represent a promising avenue for generation of more resistant crop plants.

## Author contributions

NA together with UB designed experiments and interpreted results. NA, DB, PJ, ON, HP, and SS performed the screen on *Solanaceae* spp. and NA performed all other experiments. AB, JG, PJ, ON, HP, JS, and SS provided strains and expression constructs. JG and JS provided the *eds1* mutant line. CD performed cluster analysis. NA, ST, and UB prepared the manuscript with contribution from JS and all authors reviewed the manuscript.

## Funding

This work was funded by grants to UB from the Deutsche Forschungsgemeinschaft (CRC 648 “Molecular mechanisms of information processing in plants”) and the Bundesministerium für Bildung und Forschung (“tools, targets & therapeutics–ProNet-T3”).

### Conflict of interest statement

The authors declare that the research was conducted in the absence of any commercial or financial relationships that could be construed as a potential conflict of interest.

## References

[B1] Aarts N. Metz M. Holub E. Staskawicz B. J. Daniels M. J. Parker J. E. (1998). Different requirements for *EDS1* and *NDR1* by disease resistance genes define at least two *R* gene-mediated signaling pathways in *Arabidopsis*. Proc. Natl. Acad. Sci. U.S.A. 95, 10306–10311. 10.1073/pnas.95.17.10306 9707643 PMC21504

[B2] Andolfo G. Sanseverino W. Rombauts S. Van de Peer Y. Bradeen J. M. Carputo D.. (2013). Overview of tomato (*Solanum lycopersicum*) candidate pathogen recognition genes reveals important *Solanum* R locus dynamics. New Phytol. 197, 223–237. 10.1111/j.1469-8137.2012.04380.x 23163550

[B3] Bent A. (2016). Resistance from relatives. Nat. Biotechnol. 34, 620–621. 10.1038/nbt.3591 27281420

[B4] Boch J. Bonas U. Lahaye T. (2014). TAL effectors–pathogen strategies and plant resistance engineering. New Phytol. 204, 823–832. 10.1111/nph.13015 25539004

[B5] Bonas U. Schulte R. Fenselau S. Minsavage G. V. Staskawicz B. J. Stall R. E. (1991). Isolation of a gene cluster from *Xanthomonas campestris* pv. *vesicatoria* that determines pathogenicity and the hypersensitive response on pepper and tomato. Mol. Plant Microbe Interact. 4, 88.

[B6] Bonas U. Stall R. E. Staskawicz B. (1989). Genetic and structural characterization of the avirulence gene *avrBs3* from *Xanthomonas campestris* pv. *vesicatoria*. Mol. Gen. Genet. 218, 127–136. 2550761 10.1007/BF00330575

[B7] Buckley J. Kilbride E. Cevik V. Vicente J. G. Holub E. B. Mable B. K. (2016). *R*-gene variation across *Arabidopsis lyrata* subspecies: effects of population structure, selection and mating system. BMC Evol. Biol. 16:93. 10.1186/s12862-016-0665-5 27150007 PMC4858910

[B8] Buscaill P. Rivas S. (2014). Transcriptional control of plant defence responses. Curr. Opin. Plant Biol. 20, 35–46. 10.1016/j.pbi.2014.04.004 24840291

[B9] Büttner D. (2016). Behind the lines–actions of bacterial type III effector proteins in plant cells. FEMS Microbiol. Rev. 40, 894–937. 10.1093/femsre/fuw026 PMC5091034 28201715

[B10] Büttner D. He S. Y. (2009). Type III protein secretion in plant pathogenic bacteria. Plant Physiol. 150, 1656–1664. 10.1104/pp.109.139089 19458111 PMC2719110

[B11] Cesari S. Bernoux M. Moncuquet P. Kroj T. Dodds P. N. (2014). A novel conserved mechanism for plant NLR protein pairs: the “integrated decoy” hypothesis. Front. Plant Sci. 5:606. 10.3389/fpls.2014.00606 25506347 PMC4246468

[B12] Chase M. W. Knapp S. Cox A. V. Clarkson J. J. Butsko Y. Joseph J.. (2003). Molecular systematics, GISH and the origin of hybrid taxa in *Nicotiana* (Solanaceae). Ann. Bot. 92, 107–127. 10.1093/aob/mcg087 12824072 PMC4243627

[B13] Cheong M. S. Kirik A. Kim J.-G. Frame K. Kirik V. Mudgett M. B. (2014). AvrBsT acetylates *Arabidopsis* ACIP1, a protein that associates with microtubules and is required for immunity. PLoS Pathog. 10:e1003952. 10.1371/journal.ppat.1003952 24586161 PMC3930583

[B14] Clarkson J. J. Kelly L. J. Leitch A. R. Knapp S. Chase M. W. (2010). Nuclear glutamine synthetase evolution in *Nicotiana*: phylogenetics and the origins of allotetraploid and homoploid (diploid) hybrids. Mol. Phylogenet. Evol. 55, 99–112. 10.1016/j.ympev.2009.10.003 19818862

[B15] Cui H. Tsuda K. Parker J. E. (2015). Effector-triggered immunity: from pathogen perception to robust defense. Annu. Rev. Plant Biol. 66, 487–511. 10.1146/annurev-arplant-050213-040012 25494461

[B16] Daniels M. J. Barber C. E. Turner P. C. Sawczyc M. K. Byrde R. J. Fielding A. H. (1984). Cloning of genes involved in pathogenicity of *Xanthomonas campestris* pv. *campestris* using the broad host range cosmid pLAFR1. EMBO J. 3, 3323. 16453595 10.1002/j.1460-2075.1984.tb02298.x PMC557857

[B17] Eitas T. K. Nimchuk Z. L. Dangl J. L. (2008). *Arabidopsis* TAO1 is a TIR-NB-LRR protein that contributes to disease resistance induced by the *Pseudomonas syringae* effector AvrB. Proc. Natl. Acad. Sci. U.S.A. 105, 6475–6480. 10.1073/pnas.0802157105 18424557 PMC2327211

[B18] Engler C. Kandzia R. Marillonnet S. (2008). A one pot, one step, precision cloning method with high throughput capability. PLoS ONE 3:e3647. 10.1371/journal.pone.0003647 18985154 PMC2574415

[B19] Engler C. Youles M. Gruetzner R. Ehnert T.-M. Werner S. Jones J. D.. (2014). A golden gate modular cloning toolbox for plants. ASC Synth. Biol. 3, 839–843. 10.1021/sb4001504 24933124

[B20] Escolar L. Van Den Ackerveken G. Pieplow S. Rossier O. Bonas U. (2001). Type III secretion and *in planta* recognition of the *Xanthomonas* avirulence proteins AvrBs1 and AvrBsT. Mol. Plant Pathol. 2, 287–296. 10.1046/j.1464-6722.2001.00077.x 20573017

[B21] Fan J. Doerner P. (2012). Genetic and molecular basis of nonhost disease resistance: complex, yes; silver bullet, no. Curr. Opin. Plant Biol. 15, 400–406. 10.1016/j.pbi.2012.03.001 22445191

[B22] Ferrante P. Clarke C. R. Cavanaugh K. A. Michelmore R. W. Buonaurio R. Vinatzer B. A. (2009). Contributions of the effector gene *hopQ1-1* to differences in host range between *Pseudomonas syringae* pv. *phaseolicola* and *P. syringae pv. tabaci*. Mol. Plant Pathol. 10, 837–842. 10.1111/J.1364-3703.2009.00577.X 19849789 PMC6640246

[B23] Figurski D. H. Helinski D. R. (1979). Replication of an origin-containing derivative of plasmid RK2 dependent on a plasmid function provided in *trans*. Proc. Natl. Acad. Sci. U.S.A. 76, 1648–1652. 377280 10.1073/pnas.76.4.1648 PMC383447

[B24] Gill U. S. Lee S. Mysore K. S. (2015). Host versus nonhost resistance: distinct wars with similar arsenals. Phytopathology 105, 580–587. 10.1094/PHYTO-11-14-0298-RVW 25626072

[B25] Heath M. C. (2000). Nonhost resistance and nonspecific plant defenses. Curr. Opin. Plant Biol. 3, 315–319. 10.1016/S1369-5266(00)00087-X 10873843

[B26] Henry E. Yadeta K. A. Coaker G. (2013). Recognition of bacterial plant pathogens: local, systemic and transgenerational immunity. New Phytol. 199, 908–915. 10.1111/nph.12214 23909802 PMC3740753

[B27] Hu G. deHart A. K. Li Y. Ustach C. Handley V. Navarre R.. (2005). *EDS1* in tomato is required for resistance mediated by TIR-class *R* genes and the receptor-like *R* gene *Ve*. Plant J. 42, 376–391. 10.1111/j.1365-313X.2005.02380.x 15842623

[B28] Jones J. D. Dangl J. L. (2006). The plant immune system. Nature 444, 323–329. 10.1038/nature05286 17108957

[B29] Jupe F. Pritchard L. Etherington G. J. Mackenzie K. Cock P. J. Wright F.. (2012). Identification and localisation of the NB-LRR gene family within the potato genome. BMC Genomics 13:75. 10.1186/1471-2164-13-75 22336098 PMC3297505

[B30] Kadota Y. Shirasu K. Zipfel C. (2015). Regulation of the NADPH oxidase RBOHD during plant immunity. Plant Cell Physiol. 56, 1472–1480. 10.1093/pcp/pcv063 25941234

[B31] Karimi M. Inzé D. Depicker A. (2002). GATEWAY™ vectors for *Agrobacterium*-mediated plant transformation. Trends Plant Sci. 7, 193–195. 10.1016/S1360-1385(02)02251-3 11992820

[B32] Kay S. Hahn S. Marois E. Hause G. Bonas U. (2007). A bacterial effector acts as a plant transcription factor and induces a cell size regulator. Science 318, 648–651. 10.1126/science.1144956 17962565

[B33] Kearney B. Staskawicz B. J. (1990). Widespread distribution and fitness contribution of *Xanthomonas campestris* avirulence gene *avrBs2*. Nature 346, 385–386. 2374611 10.1038/346385a0

[B34] Kelly L. J. Leitch A. R. Clarkson J. J. Knapp S. Chase M. W. (2013). Reconstructing the complex evolutionary origin of wild allopolyploid tobaccos (*Nicotiana* section *Suaveolentes*). Evolution 67, 80–94. 10.1111/j.1558-5646.2012.01748.x 23289563

[B35] Khan M. Subramaniam R. Desveaux D. (2016). Of guards, decoys, baits and traps: pathogen perception in plants by type III effector sensors. Curr. Opin. Microbiol. 29, 49–55. 10.1016/j.mib.2015.10.006 26599514

[B36] Kim J.-G. Stork W. Mudgett M. B. (2013). *Xanthomonas* type III effector XopD desumoylates tomato transcription factor SlERF4 to suppress ethylene responses and promote pathogen growth. Cell Host Microbe 13, 143–154. 10.1016/j.chom.2013.01.006 23414755 PMC3622456

[B37] Kim N. H. Choi H. W. Hwang B. K. (2010). Xanthomonas campestris pv. *vesicatoria* effector AvrBsT induces cell death in pepper, but suppresses defense responses in tomato. Mol. Plant Microbe Interact. 23, 1069–1082. 10.1094/MPMI-23-8-1069 20615117

[B38] Klement Z. Goodman R. (1967). The hypersensitive reaction to infection by bacterial plant pathogens. Annu. Rev. Phytopathol. 5, 17–44.

[B39] Knapp S. Chase M. W. Clarkson J. J. (2004). Nomenclatural changes and a new sectional classification in *Nicotiana* (Solanaceae). Taxon 53, 73–82. 10.2307/4135490

[B40] Koncz C. Schell J. (1986). The promoter of TL-DNA gene *5* controls the tissue-specific expression of chimaeric genes carried by a novel type of *Agrobacterium* binary vector. Mol. Gen. Genet. 204, 383–396.

[B41] Kovach M. E. Elzer P. H. Hill D. S. Robertson G. T. Farris M. A. Roop R. M.. (1995). Four new derivatives of the broad-host-range cloning vector pBBR1MCS, carrying different antibiotic-resistance cassettes. Gene 166, 175–176. 8529885 10.1016/0378-1119(95)00584-1

[B42] Lee S. Whitaker V. M. Hutton S. F. (2016). Mini review: potential applications of non-host resistance for crop improvement. Front. Plant Sci. 7:997. 10.3389/fpls.2016.00997 27462329 PMC4939297

[B43] Leitch I. J. Hanson L. Lim K. Y. Kovarik A. Chase M. W. Clarkson J. J.. (2008). The ups and downs of genome size evolution in polyploid species of *Nicotiana* (Solanaceae). Ann. Bot. 101, 805–814. 10.1093/aob/mcm326 18222910 PMC2710205

[B44] Li W. Chiang Y. H. Coaker G. (2013a). The HopQ1 effector's nucleoside hydrolase-like domain is required for bacterial virulence in arabidopsis and tomato, but not host recognition in tobacco. PLoS ONE 8:e59684 10.1371/journal.pone.0059684 23555744 PMC3608555

[B45] Li W. Yadeta K. A. Elmore J. M. Coaker G. (2013b). The *Pseudomonas syringae* effector HopQ1 promotes bacterial virulence and interacts with tomato 14-3-3 proteins in a phosphorylation-dependent manner. Plant Physiol. 161, 2062–2074. 10.1104/pp.112.211748 23417089 PMC3613476

[B46] Li X. Kapos P. Zhang Y. (2015). NLRs in plants. Curr. Opin. Immunol. 32, 114–121. 10.1016/j.coi.2015.01.014 25667191

[B47] Lindeberg M. Cunnac S. Collmer A. (2009). The evolution of Pseudomonas syringae host specificity and type III effector repertoires. Mol. Plant Pathol. 10, 767–775. 10.1111/J.1364-3703.2009.00587.X 19849783 PMC6640529

[B48] Lindeberg M. Cunnac S. Collmer A. (2012). *Pseudomonas syringae* type III effector repertoires: last words in endless arguments. Trends Microbiol. 20, 199–208. 10.1016/j.tim.2012.01.003 22341410

[B49] Lindeberg M. Stavrinides J. Chang J. H. Alfano J. R. Collmer A. Dangl J. L.. (2005). Proposed guidelines for a unified nomenclature and phylogenetic analysis of type III Hop effector proteins in the plant pathogen *Pseudomonas syringae*. Mol. Plant Microbe Interact. 18, 275–282. 10.1094/MPMI-18-0275 15828679

[B50] Lorenz C. Büttner D. (2009). Functional characterization of the type III secretion ATPase HrcN from the plant pathogen *Xanthomonas campestris* pv. vesicatoria. J. Bacteriol. 191, 1414–1428. 10.1128/JB.01446-08 19114489 PMC2648192

[B51] Luo S. Zhang Y. Hu Q. Chen J. Li K. Lu C.. (2012). Dynamic nucleotide-binding site and leucine-rich repeat-encoding genes in the grass family. Plant Physiol. 159, 197–210. 10.1104/pp.111.192062 22422941 PMC3375961

[B52] Maekawa T. Kufer T. A. Schulze-Lefert P. (2011). NLR functions in plant and animal immune systems: so far and yet so close. Nat. Immunol. 12, 817–826. 10.1038/ni.2083 21852785

[B53] Ménard R. Sansonetti P. J. Parsot C. (1993). Nonpolar mutagenesis of the ipa genes defines IpaB, IpaC, and IpaD as effectors of *Shigella flexneri* entry into epithelial cells. J. Bacteriol. 175, 5899–5906. 8376337 10.1128/jb.175.18.5899-5906.1993 PMC206670

[B54] Meng X. Zhang S. (2013). MAPK cascades in plant disease resistance signaling. Annu. Rev. Phytopathol. 51, 245–266. 10.1146/annurev-phyto-082712-102314 23663002

[B55] Metz M. Dahlbeck D. Morales C. Q. Al Sady B. Clark E. T. Staskawicz B. J. (2005). The conserved *Xanthomonas campestris* pv. *vesicatoria* effector protein XopX is a virulence factor and suppresses host defense in Nicotiana benthamiana. Plant J. 41, 801–814. 10.1111/j.1365-313X.2005.02338.x 15743446

[B56] Minsavage G. Dahlbeck D. Whalen M. Kearney B. Bonas U. Staskawicz B. (1990). Gene-for-gene relationships specifying disease resistance in *Xanthomonas campestris* pv. *vesicatoria* - pepper interactions. Mol. Plant Microbe Interact. 3, 41–47.

[B57] Nakagawa T. Kurose T. Hino T. Tanaka K. Kawamukai M. Niwa Y.. (2007). Development of series of Gateway binary vectors, pGWBs, for realizing efficient construction of fusion genes for plant transformation. J. Biosci. Bioeng. 104, 34–41. 10.1263/jbb.104.34 17697981

[B58] Niks R. E. Marcel T. C. (2009). Nonhost and basal resistance: how to explain specificity? New Phytol. 182, 817–828. 10.1111/j.1469-8137.2009.02849.x 19646067

[B59] Noël L. Thieme F. Gäbler J. Büttner D. Bonas U. (2003). XopC and XopJ, two novel type III effector proteins from *Xanthomonas campestris* pv. vesicatoria. J. Bacteriol. 185, 7092–7102. 10.1128/JB.185.24.7092-7102.2003 14645268 PMC296255

[B60] Noël L. Thieme F. Nennstiel D. Bonas U. (2001). cDNA-AFLP analysis unravels a genome-wide *hrpG*-regulon in the plant pathogen *Xanthomonas campestris* pv. *vesicatoria*. Mol. Microbiol. 41, 1271–1281. 10.1046/j.1365-2958.2001.02567.x 11580833

[B61] Ordon J. Gantner J. Kemna J. Schwalgun L. Reschke M. Streubel J.. (2016). Generation of chromosomal deletions in dicotyledonous plants employing a user-friendly genome editing toolkit. Plant J. [Epub ahead of print]. 10.1111/tpj.13319 27579989

[B62] Peart J. R. Cook G. Feys B. J. Parker J. E. Baulcombe D. C. (2002). An *EDS1* orthologue is required for *N*-mediated resistance against tobacco mosaic virus. Plant J. 29, 569–579. 10.1046/j.1365-313X.2002.029005569.x 11874570

[B63] Popov G. Fraiture M. Brunner F. Sessa G. (2016). Multiple *Xanthomonas euvesicatoria* Type III Effectors Inhibit flg22-Triggered Immunity. Mol. Plant Microbe Interact. 29, 651–660. 10.1094/MPMI-07-16-0137-R 27529660

[B64] Potnis N. Krasileva K. Chow V. Almeida N. F. Patil P. B. Ryan R. P.. (2011). Comparative genomics reveals diversity among xanthomonads infecting tomato and pepper. BMC Genomics 12:146. 10.1186/1471-2164-12-146 21396108 PMC3071791

[B65] Potnis N. Minsavage G. Smith J. K. Hurlbert J. C. Norman D. Rodrigues R.. (2012). Avirulence proteins AvrBs7 from *Xanthomonas gardneri* and AvrBs1. 1 from *Xanthomonas euvesicatoria* contribute to a novel gene-for-gene interaction in pepper. Mol. Plant Microbe Interact. 25, 307–320. 10.1094/MPMI-08-11-0205 22112215

[B66] Quirin E. A. Mann H. Meyer R. S. Traini A. Chiusano M. L. Litt A.. (2012). Evolutionary meta-analysis of Solanaceous resistance gene and *Solanum* resistance gene analog sequences and a practical framework for cross-species comparisons. Mol. Plant Microbe Interact. 25, 603–612. 10.1094/MPMI-12-11-0318-R 22352721

[B67] Roden J. A. Belt B. Ross J. B. Tachibana T. Vargas J. Mudgett M. B. (2004). A genetic screen to isolate type III effectors translocated into pepper cells during *Xanthomonas* infection. Proc. Natl. Acad. Sci. U.S.A. 101, 16624–16629. 10.1073/pnas.0407383101 15545602 PMC534543

[B68] Römer P. Hahn S. Jordan T. Strauss T. Bonas U. Lahaye T. (2007). Plant pathogen recognition mediated by promoter activation of the pepper *Bs3* resistance gene. Science 318, 645–648. 10.1126/science.1144958 17962564

[B69] Ronald P. C. Staskawicz B. J. (1988). The avirulence gene *avrBs1* from *Xanthomonas campestris* pv. *vesicatoria* encodes a 50-kD protein. Mol. Plant Microbe Interact. 1, 191–198. 2979910

[B70] Salomon D. Dar D. Sreeramulu S. Sessa G. (2011). Expression of *Xanthomonas campestris* pv. *vesicatoria* type III effectors in yeast affects cell growth and viability. Mol. Plant Microbe Interact. 24, 305–314. 10.1094/MPMI-09-10-0196 21062109

[B71] Schreiber T. Sorgatz A. List F. Blüher D. Thieme S. Wilmanns M.. (2015). Refined requirements for protein regions important for activity of the TALE AvrBs3. PLoS ONE 10:e0120214. 10.1371/journal.pone.0120214 25781334 PMC4363659

[B72] Schulze S. Kay S. Büttner D. Egler M. Eschen-Lippold L. Hause G.. (2012). Analysis of new type III effectors from *Xanthomonas* uncovers XopB and XopS as suppressors of plant immunity. New Phytol. 195, 894–911. 10.1111/j.1469-8137.2012.04210.x 22738163

[B73] Schwartz A. R. Potnis N. Timilsina S. Wilson M. Patané J. Martins J. Jr.. (2015). Phylogenomics of *Xanthomonas* field strains infecting pepper and tomato reveals diversity in effector repertoires and identifies determinants of host specificity. Front. Microbiol. 6:535. 10.3389/fmicb.2015.00535 26089818 PMC4452888

[B74] Schwessinger B. Ronald P. C. (2012). Plant innate immunity: perception of conserved microbial signatures. Annu. Rev. Plant Biol. 63, 451–482. 10.1146/annurev-arplant-042811-105518 22404464

[B75] Senthil-Kumar M. Mysore K. S. (2013). Nonhost resistance against bacterial pathogens: retrospectives and prospects. Annu. Rev. Phytopathol. 51, 407–427. 10.1146/annurev-phyto-082712-102319 23725473

[B76] Sierro N. Battey J. N. D. Ouadi S. Bakaher N. Bovet L. Willig A.. (2014). The tobacco genome sequence and its comparison with those of tomato and potato. Nat. Commun. 5:3833. 10.1038/ncomms4833 24807620 PMC4024737

[B77] Sierro N. Battey J. N. Ouadi S. Bovet L. Goepfert S. Bakaher N.. (2013). Reference genomes and transcriptomes of *Nicotiana sylvestris* and *Nicotiana tomentosiformis*. Genome Biol. 14:R60. 10.1186/gb-2013-14-6-r60 23773524 PMC3707018

[B78] Singer A. U. Schulze S. Skarina T. Xu X. Cui H. Eschen-Lippold L.. (2013). A pathogen type III effector with a novel E3 ubiquitin ligase architecture. PLoS Pathog. 9:e1003121. 10.1371/journal.ppat.1003121 23359647 PMC3554608

[B79] Sohn K. H. Zhang Y. Jones J. D. (2009). The *Pseudomonas syringae* effector protein, AvrRPS4, requires *in planta* processing and the KRVY domain to function. Plant J. 57, 1079–1091. 10.1111/j.1365-313X.2008.03751.x 19054367

[B80] Stall R. E. (1995). Xanthomonas campestris pv. vesicatoria, in Pathogenesis and Host-Parasite Specificity in Plant Diseases, eds Singh R. P. S. U. S. Kohmoto K. (Tarrytown, NY: Pergamon, Elsevier Science Inc.), 167–184.

[B81] Stork W. Kim J.-G. Mudgett M. B. (2015). Functional analysis of plant defense suppression and activation by the *Xanthomonas* core type III effector XopX. Mol. Plant Microbe Interact. 28, 180–194. 10.1094/MPMI-09-14-0263-R 25338145 PMC4293322

[B82] Sukarta O. C. Slootweg E. J. Goverse A. (2016). Structure-informed insights for NLR functioning in plant immunity. Semin. Cell Dev. Biol. 56, 134–149. 10.1016/j.semcdb.2016.05.012 27208725

[B83] Szczesny R. Büttner D. Escolar L. Schulze S. Seiferth A. Bonas U. (2010a). Suppression of the AvrBs1-specific hypersensitive response by the YopJ effector homolog AvrBsT from *Xanthomonas* depends on a SNF1-related kinase. New Phytol. 187, 1058–1074. 10.1111/j.1469-8137.2010.03346.x 20609114

[B84] Szczesny R. Jordan M. Schramm C. Schulz S. Cogez V. Bonas U.. (2010b). Functional characterization of the Xcs and Xps type II secretion systems from the plant pathogenic bacterium *Xanthomonas campestris* pv. *vesicatoria*. New Phytol. 187, 983–1002. 10.1111/j.1469-8137.2010.03312.x 20524995

[B85] Takken F. L. Goverse A. (2012). How to build a pathogen detector: structural basis of NB-LRR function. Curr. Opin. Plant Biol. 15, 375–384. 10.1016/j.pbi.2012.05.001 22658703

[B86] Teper D. Burstein D. Salomon D. Gershovitz M. Pupko T. Sessa G. (2016). Identification of novel *Xanthomonas euvesicatoria* type III effector proteins by a machine-learning approach. Mol. Plant Pathol. 17, 398–411. 10.1111/mpp.12288 26104875 PMC6638362

[B87] Teper D. Salomon D. Sunitha S. Kim J. G. Mudgett M. B. Sessa G. (2014). *Xanthomonas euvesicatoria* type III effector XopQ interacts with tomato and pepper 14-3-3 isoforms to suppress effector-triggered immunity. Plant J. 77, 297–309. 10.1111/tpj.12391 24279912

[B88] Thieme F. Koebnik R. Bekel T. Berger C. Boch J. Büttner D.. (2005). Insights into genome plasticity and pathogenicity of the plant pathogenic bacterium *Xanthomonas campestris* pv. *vesicatoria* revealed by the complete genome sequence. J. Bacteriol. 187, 7254–7266. 10.1128/JB.187.21.7254-7266.2005 16237009 PMC1272972

[B89] Thieme F. Szczesny R. Urban A. Kirchner O. Hause G. Bonas U. (2007). New type III effectors from Xanthomonas campestris pv. *vesicatoria* trigger plant reactions dependent on a conserved N-myristoylation motif. Mol. Plant Microbe Interact. 20, 1250–1261. 10.1094/MPMI-20-10-1250 17918627

[B90] Thordal-Christensen H. (2003). Fresh insights into processes of nonhost resistance. Curr. Opin. Plant Biol. 6, 351–357. 10.1016/S1369-5266(03)00063-3 12873530

[B91] Uma B. Rani T. S. Podile A. R. (2011). Warriors at the gate that never sleep: non-host resistance in plants. J. Plant Physiol. 168, 2141–2152. 10.1016/j.jplph.2011.09.005 22001579

[B92] Üstün S. Bartetzko V. Börnke F. (2013). The *Xanthomonas campestris* type III effector XopJ targets the host cell proteasome to suppress salicylic-acid mediated plant defence. PLoS Pathog. 9:e1003427. 10.1371/journal.ppat.1003427 23785289 PMC3681735

[B93] Wei C. Chen J. Kuang H. (2016). Dramatic Number Variation of *R* Genes in Solanaceae Species Accounted for by a Few *R* Gene Subfamilies. PLoS ONE 11:e0148708. 10.1371/journal.pone.0148708 26849045 PMC4743996

[B94] Wei C. F. Kvitko B. H. Shimizu R. Crabill E. Alfano J. R. Lin N. C.. (2007). A *Pseudomonas syringae* pv. *tomato* DC3000 mutant lacking the type III effector HopQ1-1 is able to cause disease in the model plant *Nicotiana benthamiana*. Plant J. 51, 32–46. 10.1111/j.1365-313X.2007.03126.x 17559511

[B95] Whalen M. C. Wang J. F. Carland F. M. Heiskell M. E. Dahlbeck D. Minsavage G. V.. (1993). Avirulence gene *avrRxv* from *Xanthomonas campestris* pv. *vesicatoria* specifies resistance on tomato line Hawaii 7998. Mol. Plant Microbe Interact. 6, 616–627. 8274773 10.1094/mpmi-6-616

[B96] Whalen M. Richter T. Zakhareyvich K. Yoshikawa M. Al-Azzeh D. Adefioye A.. (2008). Identification of a host 14-3-3 Protein that Interacts with *Xanthomonas* effector *AvrRxv*. Physiol. Mol. Plant Pathol. 72, 46–55. 10.1016/j.pmpp.2008.05.006 21796232 PMC3142867

[B97] Wirthmueller L. Zhang Y. Jones J. D. Parker J. E. (2007). Nuclear accumulation of the *Arabidopsis* immune receptor RPS4 is necessary for triggering EDS1-dependent defense. Curr. Biol. 17, 2023–2029. 10.1016/j.cub.2007.10.042 17997306

[B98] Wroblewski T. Caldwell K. S. Piskurewicz U. Cavanaugh K. A. Xu H. Kozik A.. (2009). Comparative large-scale analysis of interactions between several crop species and the effector repertoires from multiple pathovars of *Pseudomonas* and *Ralstonia*. Plant Physiol. 150, 1733–1749. 10.1104/pp.109.140251 19571308 PMC2719141

[B99] Yang S. Gu T. Pan C. Feng Z. Ding J. Hang Y.. (2008). Genetic variation of NBS-LRR class resistance genes in rice lines. Theor. Appl. Genet. 116, 165–177. 10.1007/s00122-007-0656-4 17932646

[B100] Yang S. Li J. Zhang X. Zhang Q. Huang J. Chen J.-Q.. (2013). Rapidly evolving *R* genes in diverse grass species confer resistance to rice blast disease. Proc. Natl. Acad. Sci. U.S.A. 110, 18572–18577. 10.1007/s00122-007-0656-4 24145399 PMC3831948

[B101] Zhang R. Murat F. Pont C. Langin T. Salse J. (2014). Paleo-evolutionary plasticity of plant disease resistance genes. BMC Genomics 15:187. 10.1186/1471-2164-15-187 24617999 PMC4234491

[B102] Zhao B. Dahlbeck D. Krasileva K. V. Fong R. W. Staskawicz B. J. (2011). Computational and biochemical analysis of the *Xanthomonas* effector AvrBs2 and its role in the modulation of *Xanthomonas* type three effector delivery. PLoS Pathog. 7:e1002408. 10.1371/journal.ppat.1002408 22144898 PMC3228805

[B103] Zheng F. Wu H. Zhang R. Li S. He W. Wong F.-L.. (2016). Molecular phylogeny and dynamic evolution of disease resistance genes in the legume family. BMC Genomics 17:402. 10.1186/s12864-016-2736-9 27229309 PMC4881053

