# Supplementary material for: Non-host Resistance Induced by the Xanthomonas Effector XopQ Is Widespread within the Genus Nicotiana and Functionally Depends on EDS1
Source: Front Plant Sci. 2016 Nov 30;7:1796. doi: 10.3389/fpls.2016.01796 (PMC5127841; doi:10.3389/fpls.2016.01796)
Supplement: Supplementary file 1 [file Table1.docx]

**Table S1: Plasmids and vectors used in this study**

| **Plasmid** | **Relevant characteristics** | **Reference** |
| --- | --- | --- |
| pBRM | Golden Gate cloning, derivative of pBBR1MCS-5, expression in *Xcv*, *lac* promoter | (Szczesny et al., 2010) |
| pBRM:xopQ | Derivative of pBRM encoding *xopQ::c-Myc* | This study |
| pENTR/D-TOPO | Entry vector for Gateway cloning | Thermo Fisher Scientific |
| pENTR/D:avrBsT | Derivative of pENTR/D-TOPO encoding *avrBsT* | This study |
| pENTR/D:avrRxv | Derivative of pENTR/D-TOPO encoding *avrRxv* | This study |
| pENTR/D:xopC | Derivative of pENTR/D-TOPO encoding *xopC* | This study |
| pENTR/D:xopH | Derivative of pENTR/D-TOPO encoding *xopH* | This study |
| pGGA1 | Golden Gate cloning, derivative of pBGWFS7; binary expression vector; *35S* promoter-Cm-*ccd*B-*GFP* | (Schulze et al., 2012) |
| pGGA1:xopO | Derivative of pGGA1 encoding *xopO::GFP* | This study |
| pGGA1:xopP | Derivative of pGGA1 encoding *xopP::GFP* | This study |
| pGGA1:xopQ | Derivative of pGGA1 encoding *xopQ::GFP* | This study |
| pGGA2 | Golden Gate cloning, derivative of pBGWFS7; binary expression vector; *35S* promoter*-GFP*-Cm-*ccd*B | (Schreiber et al., 2015) |
| pGGA2:xopG | Derivative of pGGA2 encoding *GFP::xopG* | This study |
| pGGA7 | Golden Gate cloning, derivative of pBGWFS7; binary expression vector; *35S* promoter-Cm-*ccd*B-4×c-Myc | This study |
| pGGA7:GFP | Derivative of pGGA7 encoding *GFP::c-Myc* | This study |
| pGGX1 | Golden Gate cloning, derivative of pBBR1MCS-5; expression in *Xcv*, *lac* promoter-Cm-*ccd*B-FLAG | This study |
| pGGX1:xopC | Derivative of pGGX1 encoding *xopC::FLAG* | This study |
| pGWB2:avrBs2 | Derivative of pGWB2 encoding *avrBs2* | (Schulze et al., 2012) |
| pGWB2:xopL_strep | Derivative of pGWB2 encoding *xopL::Strep* | (Singer et al., 2013) |
| pGWB5 | Gateway cloning, binary expression vector; *35S* promoter-*att*R1-Cm-*ccd*B-*attR*2-*GFP* | (Nakagawa et al., 2007) |
| pGWB5:avrBs1 | Derivative of pGWB5 encoding *avrBs1::GFP* | (Guerlebeck et al., 2009) |
| pGWB5:avrBs3 | Derivative of pGWB5 encoding *avrBs3::GFP* | (Morbitzer et al., 2011) |
| pGWB5:avrRxv | Derivative of pGWB5 encoding *avrRxv::GFP* | This study |
| pGWB5:xopJ | Derivative of pGWB5 encoding *xopJ::GFP* | (Thieme et al., 2007) |
| pGWB5:xopK | Derivative of pGWB5 encoding *xopK::GFP* | (Schulze et al., 2012) |
| pGWB5:xopM | Derivative of pGWB5 encoding *xopM::GFP* | (Schulze et al., 2012) |
| pGWB5:xopV | Derivative of pGWB5 encoding *xopV::GFP* | (Schulze et al., 2012) |
| pGWB6 | Gateway cloning, binary expression vector; *35S* promoter-*att*R1-*GFP*-*Cm*-*ccd*B-*attR*2 | (Nakagawa et al., 2007) |
| pGWB6:avrBsT | Derivative of pGWB6 encoding *GFP::avrBsT* | This study |
| pJET1.2/blunt | Classical cloning vector | Thermo Fisher Scientific |
| pJET1.2/blunt:xopP | Derivative of pJETpJET1.2/blunt encoding *xopP* | This study |
| pJET1.2/blunt:xopO | Derivative of pJETpJET1.2/blunt encoding *xopO* | This study |
| pK7FWG2 | Gateway cloning, *35S* promoter, C-terminal fusion of GFP to gene of interest | (Karimi et al., 2002) |
| pK7FWG2:xopB | Derivative of pK7FWG2encoding *xopB::GFP* | (Schulze et al., 2012) |
| pK7FWG2:xopC | Derivative of pK7FWG2encoding *xopC::GFP* | This study |
| pK7FWG2:xopE1 | Derivative of pK7FWG2encoding *xopE1::GFP* | (Thieme et al., 2007) |
| pK7FWG2:xopE2 | Derivative of pK7FWG2encoding *xopE2::GFP* | (Thieme et al., 2007) |
| pK7FWG2:xopH | Derivative of pK7FWG2 encoding *xopH::GFP* | This study |
| pK7FWG2:xopI | Derivative of pK7FWG2 encoding *xopI::GFP* | (Schulze et al., 2012) |
| pK7FWG2:xopS | Derivative of pK7FWG2 encoding *xopS::GFP* | (Schulze et al., 2012) |
| pOGG2 | Golden Gate cloning, derivative of suicide vector pOK1, *sacB* *sacQ* *mobRK1* oriR6K | (Schulze et al., 2012) |
| pOGG2:xopC_fl | Derivative of pOGG2 carrying flanking sequences and 5’ and 3’ sequences of *xopC* | This study |
| pOGG2:xopQ_fl | Derivative of pOGG2 carrying flanking sequences of *xopQ* | This study |
| pUC57 | Classical cloning vector | Thermo Fisher Scientific |
| pUC57:xopC | Derivative of pUC57 encoding *xopC* | This study |
| pUC57:xopQ | Derivative of pUC57 encoding *xopQ* | This study |
| pAGM1287 | Component of Golden Gate Modular Cloning Toolbox for Plants; level zero acceptor for CDS1 no stop modules | (Engler et al., 2014) |
| pJOG285 | Derivative of pAGM1287 containing codon-altered fragment encoding *Nb*EDS1a without stop codon | This study |
| pICH47732 | Component of Golden Gate Modular Cloning Toolbox for Plants; level 1 acceptor | (Engler et al., 2014) |
| pICH51277 | Component of Golden Gate Modular Cloning Toolbox for Plants; provides promoter module (*35S* promoter) | (Engler et al., 2014) |
| pICH41432 | Component of Golden Gate Modular Cloning Toolbox for Plants; provides terminator module | (Engler et al., 2014) |
| pICH50010 | Component of Golden Gate Modular Cloning Toolbox for Plants; provides C-terminal c-Myc module | (Engler et al., 2014) |
| pJOG296 | Assembly product from pICH47732 with pICH51277, pICH41432, pICH50010 and pJOG285 encoding *NbEDS1a::c-Myc* under *35S* promoter control | This study |

Engler, C., Youles, M., Gruetzner, R., Ehnert, T.-M., Werner, S., Jones, J.D., et al. (2014). A golden gate modular cloning toolbox for plants. *ASC Synth Biol* 3, 839-843. 10.1021/sb4001504.

Guerlebeck, D., Jahn, S., Guerlebeck, N., Szczesny, R., Szurek, B., Hahn, S., et al. (2009). Visualization of novel virulence activities of the *Xanthomonas* type III effectors AvrBs1, AvrBs3 and AvrBs4. *Mol Plant Pathol* 10, 175-188. 10.1111/J.1364-3703.2008.00519.X.

Karimi, M., Inzé, D., and Depicker, A. (2002). GATEWAY™ vectors for *Agrobacterium*-mediated plant transformation. *Trends Plant Sci* 7, 193-195.

Morbitzer, R., Elsaesser, J., Hausner, J., and Lahaye, T. (2011). Assembly of custom TALE-type DNA binding domains by modular cloning. *Nucleic Acids Res* 39, 5790-5799.

Nakagawa, T., Kurose, T., Hino, T., Tanaka, K., Kawamukai, M., Niwa, Y., et al. (2007). Development of series of gateway binary vectors, pGWBs, for realizing efficient construction of fusion genes for plant transformation. *J Biosci Bioeng* 104, 34-41. 10.1263/jbb.104.34.

Schreiber, T., Sorgatz, A., List, F., Blüher, D., Thieme, S., Wilmanns, M., et al. (2015). Refined requirements for protein regions important for activity of the TALE AvrBs3. *PloS ONE* 10, e0120214. 10.1371/journal.pone.0120214.

Schulze, S., Kay, S., Büttner, D., Egler, M., Eschen-Lippold, L., Hause, G., et al. (2012). Analysis of new type III effectors from *Xanthomonas* uncovers XopB and XopS as suppressors of plant immunity. *New Phytol* 195, 894-911. 10.1111/j.1469-8137.2012.04210.x.

Singer, A.U., Schulze, S., Skarina, T., Xu, X., Cui, H., Eschen-Lippold, L., et al. (2013). A pathogen type III effector with a novel E3 ubiquitin ligase architecture. *PLoS Pathog* 9, e1003121. 10.1371/journal.ppat.1003121.

Szczesny, R., Jordan, M., Schramm, C., Schulz, S., Cogez, V., Bonas, U., et al. (2010). Functional characterization of the Xcs and Xps type II secretion systems from the plant pathogenic bacterium *Xanthomonas campestris* pv *vesicatoria*. *New Phytol* 187, 983-1002. 10.1111/j.1469-8137.2010.03312.x.

Thieme, F., Szczesny, R., Urban, A., Kirchner, O., Hause, G., and Bonas, U. (2007). New type III effectors from *Xanthomonas campestris* pv. *vesicatoria* trigger plant reactions dependent on a conserved N-myristoylation motif. *Mol Plant Microbe Interact* 20, 1250-1261. 10.1094/MPMI-20-10-1250.
