# Supplementary material for: Non-host Resistance Induced by the Xanthomonas Effector XopQ Is Widespread within the Genus Nicotiana and Functionally Depends on EDS1
Source: Front Plant Sci. 2016 Nov 30;7:1796. doi: 10.3389/fpls.2016.01796 (PMC5127841; doi:10.3389/fpls.2016.01796)
Supplement: Supplementary file 2 [file Table2.docx]

**Table S2: Plant lines used in this study**

| **Abbreviation** | **Botanical name** | ***Nicotiana* section** | **Accession name** | **Accession number** | **Origin** | **Source** |
| --- | --- | --- | --- | --- | --- | --- |
| *Nphy 1* | *Nicandra physalodes* (L.) Gaertn. | *-* | - | NICA 1 | unknown | IPK |
| *Nphy 2* | *Nicandra physalodes* (L.) Gaertn. | *-* | bingfeng zi â | NICA 7 | China | IPK |
| *Nacu* | *Nicotiana acuminata* (Graham) Hook. | *Petunioides* | - | NIC 471 | USA | IPK |
| *Nafr* | *Nicotiana africana* Merxm. | *Suaveolentes* | - | NIC 464 | Namibia | IPK |
| *Nala* | *Nicotiana alata* "Sweet White" | *Alatae* | - | 32558 | unknown | B & T |
| *Nbena* | *Nicotiana benavidesii* Goodsp. | *Paniculatae* | - | NIC 442 | unknown | IPK |
| *Nbent* | *Nicotiana benthamiana* Domin | *Suaveolentes* | - | NIC 660 | unknown | IPK |
| *Nbon* | *Nicotiana bonariensis* Lehm. | *Alatae* | - | NIC 524 | unknown | IPK |
| *Ncle* | *Nicotiana clevelandii* A.Gray | *Polydicliae* | - | NIC 420 | unknown | IPK |
| *Nexc* | *Nicotiana excelsior* (J.M.Black) J.M.Black | *Suaveolentes* | - | NIC 400 | Australia | IPK |
| *Ngla* | *Nicotiana glauca* | *Noctiflorae* | - | 400282 | unknown | B & T |
| *Nglu* | *Nicotiana glutinosa* L. | *Undulatae* | - | NIC 419 | unknown | IPK |
| *Ning* | *Nicotiana ingulba* J.M.Black | *Suaveolentes* | - | NIC 427 | unknown | IPK |
| *Nkaw* | *Nicotiana kawakamii* Y.Ohashi | *Tomentosae* | - | NIC 486 | unknown | IPK |
| *Nlan* | *Nicotiana langsdorffii* Weinm. | *Alatae* | - | NIC 528 | unknown | IPK |
| *Nnoc* | *Nicotiana noctiflora* Hook. | *Noctiflorae* | - | NIC 476 | unknown | IPK |
| *Nnud* | *Nicotiana nudicaulis* S.Watson | *Repandae* | - | NIC 433 | unknown | IPK |
| *Npal* | *Nicotiana palmeri* A.Gray | *Trigonophyllae* | - | NIC 439 | unknown | IPK |
| *Npan* | *Nicotiana paniculata* L. | *Paniculatae* | - | NIC 435 | Peru | IPK |
| *Npau* | *Nicotiana pauciflora* J.Remy | *Petunioides* | - | NIC 454 | unknown | IPK |
| *Nplu* | *Nicotiana plumbaginifolia* Viv. | *Alatae* | - | NIC 430 | Peru | IPK |
| *Nqua* | *Nicotiana quadrivalvis* Pursh var. wallacei (A.Gray) Mansf. | *Polydicliae* | - | NIC 421 | unknown | IPK |
| *Nrep* | *Nicotiana repanda* Willd. ex Lehm. | *Repandae* | - | NIC 521 | unknown | IPK |
| *Nrot* | *Nicotiana rotundifolia* Lindl. | *Suaveolentes* | - | NIC 482 | unknown | IPK |
| *Nrus* | *Nicotiana rustica* "Midewiwan Sacred Tobacco" | *Rusticae* | - | 406310 | unknown | B & T |
| *Nsan* | *Nicotiana sanderae* "Lime Green" |  | - | 32557 | unknown | B & T |
| *Nsyl* | *Nicotiana sylvestris* Speg. & Comes | *Sylvestres* | - | NIC 6 | unknown | IPK |
| *Ntab 1* | *Nicotiana tabacum* L. var. *attenuata* Schrank | *Nicotiana* | Joiven | NIC 264 | unknown | IPK |
| *Ntab 2* | *Nicotiana tabacum* L. var. *attenuata* Schrank | *Nicotiana* | Dresden | NIC 311 | Germany | IPK |
| *Ntab 3* | *Nicotiana tabacum* L. var. *attenuata* Schrank | *Nicotiana* | Tamasesti 50 | NIC 364 | Romania | IPK |
| *Ntab 4* | *Nicotiana tabacum* L. var. *havanensis* Comes | *Nicotiana* | Orinoco (TI 81) | NIC 1020 | Indonesia | IPK |
| *Ntab 5* | *Nicotiana tabacum* L. var. *havanensis* Comes | *Nicotiana* | TI 419 | NIC 1026 | Mexico | IPK |
| *Ntab 6* | *Nicotiana tabacum* L. var. *havanensis* Comes | *Nicotiana* | Boliviano (TI 437) | NIC 1027 | USA | IPK |
| *Ntab 7* | *Nicotiana tabacum* L. var. *havanensis* Comes | *Nicotiana* | TI 579 | NIC 1035 | Ecuador | IPK |
| *Ntab 8* | *Nicotiana tabacum* L. var. *havanensis* Comes | *Nicotiana* | TI 1309 | NIC 1042 | Papua Neu Guinea | IPK |
| *Ntab 9* | *Nicotiana tabacum* L. var. *havanensis* Comes | *Nicotiana* | Baiano (TI 128) | NIC 1051 | Brazil | IPK |
| *Ntab 10* | *Nicotiana tabacum* L. var. *havanensis* Comes | *Nicotiana* | Orinoco (TI 125) | NIC 1052 | Indonesia | IPK |
| *Ntab 11* | *Nicotiana tabacum* L. var. *havanensis* Comes | *Nicotiana* | Palmira (TI 330) | NIC 1053 | Colombia | IPK |
| *Ntab 12* | *Nicotiana tabacum* L. var. *havanensis* Comes | *Nicotiana* | Dubeque (TI 1241) | NIC 1055 | Spain | IPK |
| *Ntab 13* | *Nicotiana tabacum* L. var. *havanensis* Comes | *Nicotiana* | TI 1275 | NIC 1056 | Korea | IPK |
| *Ntab 14* | *Nicotiana tabacum* L. var. *havanensis* Comes | *Nicotiana* | TI 1687 | NIC 1058 | Zambia | IPK |
| *Ntab 15* | *Nicotiana tabacum* L. var. *havanensis* Comes | *Nicotiana* | Pulawski Bialokwitnacy | NIC 239 | Poland | IPK |
| *Ntab 16* | *Nicotiana tabacum* L. var. *macrophylla* Schrank | *Nicotiana* | Sumatra Mitlingskaja | NIC 215 | Soviet Union | IPK |
| *Ntab 17* | *Nicotiana tabacum* L. var. *macrophylla* Schrank | *Nicotiana* | - | NIC 480 | Georgia | IPK |
| *Ntab 18* | *Nicotiana tabacum* L. var. *pallescens* Schrank | *Nicotiana* | Elsässer Virgin | NIC 104 | unknown | IPK |
| *Ntab 19* | *Nicotiana tabacum* L. var. *pallescens* Schrank | *Nicotiana* | Herzegowina | NIC 205 | Yugoslavia | IPK |
| *Ntab 20* | *Nicotiana tabacum* L. var. *pallescens* Schrank | *Nicotiana* | Mont Calme Brun | NIC 280 | unknown | IPK |
| *Ntab 21* | *Nicotiana tabacum* L. var. *pallescens* Schrank | *Nicotiana* | Micurinskij | NIC 366 | Soviet Union | IPK |
| *Ntab 22* | *Nicotiana tabacum* L. var. *pallescens* Schrank | *Nicotiana* | Plovdiv 34 | NIC 416 | Bulgaria | IPK |
| *Ntab 23* | *Nicotiana tabacum* L. var. *pallescens* Schrank | *Nicotiana* | pelo de oro | NIC 514 | Cuba | IPK |
| *Ntab 24* | *Nicotiana tabacum* L. var. *pallescens* Schrank | *Nicotiana* | tabaco | NIC 527 | Peru | IPK |
| *Ntab 25* | *Nicotiana tabacum* L. var. *sagittata* Danert | *Nicotiana* | Cabot | NIC 245 | unknown | IPK |
| *Ntab 26* | *Nicotiana tabacum* L. var. *sagittata* Danert | *Nicotiana* | Baragan 221 | NIC 265 | Romania | IPK |
| *Ntab 27* | *Nicotiana tabacum* L. var. *serotina* Schrank | *Nicotiana* | Kerti Kapolnai | NIC 318 | Hungary | IPK |
| *Ntab 28* | *Nicotiana tabacum* L. var. *tabacum* | *Nicotiana* | Badische Geudertheimer Landsorte | NIC 102 | Germany | IPK |
| *Ntab 29* | *Nicotiana tabacum* L. var*. tabacum* | *Nicotiana* | Jaffna (TI 501) | NIC 1030 | Guatemala | IPK |
| *Ntab 30* | *Nicotiana tabacum* L. var. *tabacum* | *Nicotiana* | Chileno Grande Amari (TI 1098) | NIC 1040 | Argentina | IPK |
| *Ntab 31* | *Nicotiana tabacum* L. var. *tabacum* | *Nicotiana* | TI 1295 | NIC 1041 | Yugoslavia | IPK |
| *Ntab 32* | *Nicotiana tabacum* L. var. *tabacum* | *Nicotiana* | Ostrollst 2747 11 (TI 1568) | NIC 1047 | Poland | IPK |
| *Ntab 33* | *Nicotiana tabacum* L. var. *tabacum* | *Nicotiana* | Correntino (TI 57) | NIC 1048 | Argentina | IPK |
| *Ntab 34* | *Nicotiana tabacum* L. var. *tabacum* | *Nicotiana* | Pina Blanca (TI 1456) | NIC 1057 | Honduras | IPK |
| *Ntab 35* | *Nicotiana tabacum* L. var. *tabacum* | *Nicotiana* | Uckermärker x U Stamm | NIC 114 | Germany | IPK |
| *Ntab 36* | *Nicotiana tabacum* L. var. *tabacum* | *Nicotiana* | Maryland | NIC 123 | USA | IPK |
| *Ntab 37* | *Nicotiana tabacum* L. var. *tabacum* | *Nicotiana* | Virginia Gold Leaf | NIC 251 | USA | IPK |
| *Ntab 38* | *Nicotiana tabacum* L. var. *tabacum* | *Nicotiana* | Virginia A | NIC 261 | Ukraine | IPK |
| *Ntab 39* | *Nicotiana tabacum* L. var. *tabacum* | *Nicotiana* | Jamaika | NIC 277 | unknown | IPK |
| *Ntab 40* | *Nicotiana tabacum* L. var. *undulata* Erh. | *Nicotiana* | Giron (TI 1113) | NIC 1038 | Venezuela | IPK |
| *Ntab 41* | *Nicotiana tabacum* L. var. *undulata* Erh | *Nicotiana* | Friedrichstaler | NIC 113 | Germany | IPK |
| *Ntab 42* | *Nicotiana tabacum* L. var. *undulata* Erh | *Nicotiana* | - | NIC 168 | Ukraine | IPK |
| *Ntab 43* | *Nicotiana tabacum* L. var. *undulata* Erh | *Nicotiana* | Havanna 322 | NIC 282 | unknown | IPK |
| *Ntab 44* | *Nicotiana tabacum* L. var. *undulata* Erh | *Nicotiana* | Greenwood | NIC 341 | unknown | IPK |
| *Ntab 45* | *Nicotiana tabacum* L. var. *undulata* Erh | *Nicotiana* | Virginia Bright Leaf | NIC 372 | USA | IPK |
| *Ntab 46* | *Nicotiana tabacum* L. var. *undulata* Erh | *Nicotiana* | - | NIC 511 | North Korea | IPK |
| *Ntom* | *Nicotiana tomentosiformis* Goodsp. | *Tomentosae* | - | NIC 479 | unknown | IPK |
| *Nvel* | *Nicotiana velutiana* H.-M.Wheeler | *Suaveolentes* | - | NIC 428 | Australia | IPK |
| *Pet* | *Petunia* spec. | *-* | - | - | unknown | internal |
| *Pper 1* | *Physalis peruviana* L. | *-* | - | PHY 35 | Rwanda | IPK |
| *Pper 2* | *Physalis peruviana* L. | *-* | - | PHY 43 | South Africa | IPK |
| *Same 1* | *Solanum americanum* Mill. | *-* | - | SOLA 424 | America | IPK |
| *Same 2* | *Solanum americanum* Mill. | *-* | - | SOLA 435 | El Salvador | IPK |
| *Snig 1* | *Solanum nigrum* L. subsp*. nigrum* var. *atriplicifolium* (Desp.) G.Mey. | *-* | - | SOLA 50 | Romania | IPK |
| *Snig 2* | *Solanum nigrum* L. subsp. *nigrum* var. *nigrum* f. humile (Bernh.) Lindm. | *-* | - | SOLA 40 | unknown | IPK |
| *Snig 3* | *Solanum nigrum* L. subsp. *nigrum* var. *nigrum* f. nigrum | *-* | - | SOLA 55 | unknown | IPK |
| *Snig 4* | *Solanum nigrum* L. subsp. *schultesii* (Opiz) Wessely | *-* | - | SOLA 294 | Austria | IPK |
| *Ssua* | *Solanum suaveolens* | *-* | - | PI 476478 01 SD | Peru | USDA |
| *Stub* | *Solanum tuberosum* | *-* | - | - | unknown | internal |

Seeds were obtained from following sources: IPK, IPK Gartersleben, Corrensstraße 3, 06466 Stadt Seeland; Germany; B & T, B & T World Seeds, Route des Marchandes, Paguignan, 34210 Aigues-Vives, France; USDA, USDA Plant Genetic Resources Conservation Unit (Georgia, USA).
