# Supplementary material for: Non-host Resistance Induced by the Xanthomonas Effector XopQ Is Widespread within the Genus Nicotiana and Functionally Depends on EDS1
Source: Front Plant Sci. 2016 Nov 30;7:1796. doi: 10.3389/fpls.2016.01796 (PMC5127841; doi:10.3389/fpls.2016.01796)
Supplement: Supplementary file 3 [file Table3.DOCX]

**Table S3: Oligonucleotides used in this study**

| **Name** | **Sequence (5´🡪3´)** | **Purpose** |
| --- | --- | --- |
| xopG_f | TTTGGTCTCATATGCCAATCAGTCAAACAAAC | Generation of pUC57:xopG |
| xopG_st_r | TTTGGTCTCTAAGCTTCACATGCCGTGAGGC | Generation of pUC57:xopG |
| xopO_f | TTAGGTCTCTTATGATCAACACTTCCGTCAAGGCG | Generation of pJET1.2/blunt:xopO |
| xopO_ns_r | TTTGGTCTCTCACCCCTGTTTATCCGACGACTTTCTTC | Generation of pJET1.2/blunt:xopO |
| xopP_f | GCGGGTCTCTTGTGCCTAAAATTGAATCGACCAAG | Generation of pJET1.2/blunt:xopP |
| xopP_ns_r | TTTGGTCTCTCACCTAGTCGAGCGCGCGCAGCGCGTC | Generation of pJET1.2/blunt:xopP |
| xopQ_f | TTTGGTCTCTTATGATGCAGCCCACCGCC | Generation of pUC57:xopQ |
| xopQ_ns_r | TTTGGTCTCTCACCGCGCCCGCGTTGC | Generation of pUC57:xopQ |
| xopQdel_5’_f | TTTGGTCTCTCGACCGCGATTACTACTCCGAAGG | Generation of pOGG2:xopQ |
| xopQdel_5’_r | TTTGGTCTCTTCCCGCGCTCCGCAGCTTTCGCG | Generation of pOGG2:xopQ |
| xopQdel_3’_f | TTTGGTCTCTGGGAGGACCTCTGGCAGTGAAAGG | Generation of pOGG2:xopQ |
| xopQdel_3’_r | TTTGGTCTCTATGGCAACCGCCGCCCAGTGCGC | Generation of pOGG2:xopQ |
| xopC_GG_f | AAAGGTCTCTTATGAAAACAAGTAGTGC | Generation of pUC57:xopC |
| xopC_GG__r | AAAGGTCTCTCACCAAGTTCCGT | Generation of pUC57:xopC |
| xopC_GW_f | CACCatgaaaacaagtagtgcaagcaag | Generation of pENTR/D:xopC |
| xopC_GW_r | aagttccgtgattttagatacaacacg | Generation of pENTR/D:xopC |
| xopCdel_5’_f | TTTGGTCTCTCGACAACCCTTTTTTGTTAAACGCGT | Generation of pOGG2:xopC |
| xopCdel_5’_r | TTTGGTCTCTGCTAAAATCTCGACCACGCTTTCCCA | Generation of pOGG2:xopC |
| xopCdel_3’_f | TTTGGTCTCTTAGCGGCAGTGACGAAGAAGCACT | Generation of pOGG2:xopC |
| xopCdel_3’_r | TTTGGTCTCTATGGAATCCCCCCATCCCCA | Generation of pOGG2:xopC |
| GFP_f | TTTGGTCTCATATGGTGAGCAAGGGCGAGGAG | Generation of pGGA7:GFP |
| GFP_ns_r | TTTGGTCTCTCACCCTTGTACAGCTCGTCCATG | Generation of pGGA7:GFP |
| avrBsT_f | CACCATGAAGAATTTTATGCGTTCAC | Generation of pENTR/D:avrBsT |
| avrBsT_r | TGATTCAATAGTTTTCCTAATTTTCCTC | Generation of pENTR/D:avrBsT |
| avrRxv_f | CACCATGTGCGACTCCATAAGAGTG | Generation of pENTR/D:avrRxv |
| avrRxv_r | GGATTCTAAGGCGTGACGGA | Generation of pENTR/D:avrRxv |
| xopH_f | CACCATGCCGAACAAAATCTC | Generation of pENTR/D:xopH |
| xopH_r | TGCATTGTGGTCGAGCCATTC | Generation of pENTR/D:xopH |

*Bsa*I-sites for Golden Gate cloning are underlined.
