# Supplementary material for: Non-host Resistance Induced by the Xanthomonas Effector XopQ Is Widespread within the Genus Nicotiana and Functionally Depends on EDS1
Source: Front Plant Sci. 2016 Nov 30;7:1796. doi: 10.3389/fpls.2016.01796 (PMC5127841; doi:10.3389/fpls.2016.01796)
Supplement: Supplementary file 5 [file Table5.docx]

**Table S5: Reactions of 18 solanaceous plant lines to *Agrobacterium*-mediated expression of *Xcv* T3Es and *Xcv* inoculation.**

| **Plant line** | **Experiment^a)^** | ***Agrobacterium*-mediated expression^b)^** | | | | | | | | | | | | | | | | | | | | | | ***Xcv* infection^c)^** | | | |
| --- | --- | --- | --- | --- | --- | --- | --- | --- | --- | --- | --- | --- | --- | --- | --- | --- | --- | --- | --- | --- | --- | --- | --- | --- | --- | --- | --- |
|  |  | **AvrBs1** | **AvrBs2** | **AvrBs3** | **AvrBsT** | **AvrRxv** | **XopB** | **XopC** | **XopE1** | **XopE2** | **XopG** | **XopH** | **XopI** | **XopJ** | **XopK** | **XopL** | **XopM** | **XopO** | **XopP** | **XopQ** | **XopS** | **XopV** | **GFP** | **85-10** | **Δ*xopQ*** | **Δ*xopQ* (p*xopQ*)** | **Δ*hrcN*** |
| *Nphy 1* | 1* | **+** | n | **+** | **+** | **+** | **+** | **+** | **-** | **+** | **+** | **+** | **+** | **+** | **-** | **+** | **+** | **+** | **-** | **+** | **+** | **+** | **+** |  |  |  |  |
|  | 2 | n | n | n | n | n | n | n | n | n | n | n | n | n | n | n | n | n | n | n | n | n | n |  |  |  |  |
|  | 3 | n | n | n | n | n | n | n | n | n | n | n | n | n | n | n | n | n | n | n | n | n | n |  |  |  |  |
| *Nacu* | 1* | **-** | n | **+** | **-** | **-** | **-** | **+** | **-** | **+** | **+** | **+** | **-** | **-** | **-** | **+** | **-** | **-** | **-** | **-** | **-** | + | + |  |  |  |  |
|  | 2 | n | n | n | n | n | n | n | n | n | n | n | n | n | n | n | n | n | n | n | n | n | n |  |  |  |  |
|  | 3 | n | n | n | n | n | n | n | n | n | n | n | n | n | n | n | n | n | n | n | n | n | n |  |  |  |  |
| *Nafr* | 1* | **+** | n | **+** | **+** | **-** | **+** | **+** | **-** | **+** | **+** | **+** | **+** | **+** | **-** | **+** | **+** | **+** | **+** | **-** | **+** | **+** | **+** |  |  |  |  |
|  | 2 | n | n | n | n | n | n | n | n | n | n | n | n | n | n | n | n | n | n | n | n | n | n |  |  |  |  |
|  | 3 | n | n | n | n | n | n | n | n | n | n | n | n | n | n | n | n | n | n | n | n | n | n |  |  |  |  |
| *Nbena* | 1* | **+** | n | **+** | **-** | **-** | **+** | **-** | **-** | **+** | **-** | **+** | **+** | **+** | **-** | **+** | **-** | **+** | **+** | **-** | **+** | **+** | **+** |  |  |  |  |
|  | 2 | n | n | n | n | n | n | n | n | n | n | n | n | n | n | n | n | n | n | n | n | n | n |  |  |  |  |
|  | 3 | n | n | n | n | n | n | n | n | n | n | n | n | n | n | n | n | n | n | n | n | n | n |  |  |  |  |
| *Ncle* | 1* | **+** | n | **-** | **-** | **+** | **+** | **+** | **-** | **+** | **+** | **+** | **+** | **+** | **-** | **+** | **+** | **+** | **+** | **+** | **+** | **+** | **+** |  |  |  |  |
|  | 2 | n | n | n | n | n | n | n | n | n | n | n | n | n | n | n | n | n | n | n | n | n | n |  |  |  |  |
|  | 3 | n | n | n | n | n | n | n | n | n | n | n | n | n | n | n | n | n | n | n | n | n | n |  |  |  |  |
| *Nexc* | 1* | **+** | n | **-** | **-** | **+** | **+** | **+** | **-** | **+** | **-** | **+** | **+** | **+** | **-** | **+** | **+** | **+** | **-** | **+** | **+** | **+** | **+** |  |  |  |  |
|  | 2 | n | n | n | n | n | n | n | n | n | n | n | n | n | n | n | n | n | n | n | n | n | n |  |  |  |  |
|  | 3 | n | n | n | n | n | n | n | n | n | n | n | n | n | n | n | n | n | n | n | n | n | n |  |  |  |  |
| *Nglu* | 1* | **+** | n | **+** | **-** | **+** | **+** | **+** | **-** | **+** | **+** | **+** | **+** | **+** | **-** | **+** | **+** | **+** | **-** | **-** | **+** | **+** | **+** |  |  |  |  |
|  | 2* | **+** | n | **+** | **-** | **-** | **+** | **+** | **-** | **+** | **+** | **+** | **+** | **+** | **-** | **+** | **+** | **+** | **+** | **+** | **+** | **+** | **+** |  |  |  |  |
|  | 3 | n | n | n | n | n | n | n | n | n | n | n | n | n | n | n | n | n | n | n | n | n | n |  |  |  |  |
|  | 4 | n | n | n | n | n | n | n | n | n | n | n | n | n | n | n | n | n | n | n | n | n | n |  |  |  |  |
| *Ning* | 1* | **+** | n | **-** | **-** | **-** | **+** | **+** | **-** | **+** | **-** | **+** | **-** | **-** | **-** | **-** | **-** | **-** | **-** | **-** | **-** | **+** | **+** |  |  |  |  |
|  | 2 | n | n | n | n | n | n | n | n | n | n | n | n | n | n | n | n | n | n | n | n | n | n |  |  |  |  |
|  | 3 | n | n | n | n | n | n | n | n | n | n | n | n | n | n | n | n | n | n | n | n | n | n |  |  |  |  |
| *Nkaw* | 1* | **+** | n | **-** | **-** | **-** | **-** | **-** | **-** | **+** | **-** | **+** | **-** | **+** | **-** | **-** | **-** | **-** | **-** | **-** | **+** | **+** | **+** |  |  |  |  |
|  | 2 | n | n | n | n | n | n | n | n | n | n | n | n | n | n | n | n | n | n | n | n | n | n |  |  |  |  |
|  | 3 | n | n | n | n | n | n | n | n | n | n | n | n | n | n | n | n | n | n | n | n | n | n |  |  |  |  |
| *Nnud* | 1* | **+** | n | **-** | **-** | **-** | **-** | **+** | **-** | **+** | **-** | **+** | **+** | **-** | **-** | **+** | **-** | **+** | **-** | **-** | **-** | **-** | **-** |  |  |  |  |
|  | 2 | n | n | n | n | n | n | n | n | n | n | n | n | n | n | n | n | n | n | n | n | n | n |  |  |  |  |
|  | 3 | n | n | n | n | n | n | n | n | n | n | n | n | n | n | n | n | n | n | n | n | n | n |  |  |  |  |
| *Npal* | 1* | **-** | n | **-** | **-** | **-** | **-** | **-** | **-** | **-** | **-** | **-** | **-** | **-** | **-** | **-** | **-** | **-** | **-** | **-** | **-** | **-** | **-** |  |  |  |  |
|  | 2 | n | n | n | n | n | n | n | n | n | n | n | n | n | n | n | n | n | n | n | n | n | n |  |  |  |  |
|  | 3 | n | n | n | n | n | n | n | n | n | n | n | n | n | n | n | n | n | n | n | n | n | n |  |  |  |  |
| *Npan* | 1* | **+** | n | **+** | **-** | **+** | **+** | **+** | **-** | **+** | **+** | **+** | **+** | **+** | **-** | **+** | **+** | **+** | **+** | **-** | **+** | **+** | **+** |  |  |  |  |
|  | 2* | **+** | n | **+** | **+** | **+** | **+** | **+** | **-** | **+** | **+** | **+** | **+** | **+** | **-** | **-** | **+** | **+** | **-** | **+** | **+** | **+** | **+** |  |  |  |  |
|  | 3 | n | n | n | n | n | n | n | n | n | n | n | n | n | n | n | n | n | n | n | n | n | n |  |  |  |  |
|  | 4 | n | n | n | n | n | n | n | n | n | n | n | n | n | n | n | n | n | n | n | n | n | n |  |  |  |  |
| *Npau* | 1* | **-** | n | **-** | **-** | **-** | **-** | **-** | **-** | **+** | **-** | **+** | **+** | **+** | **-** | **-** | **-** | **-** | **-** | **+** | **-** | **+** | **+** |  |  |  |  |
|  | 2 | n | n | n | n | n | n | n | n | n | n | n | n | n | n | n | n | n | n | n | n | n | n |  |  |  |  |
|  | 3 | n | n | n | n | n | n | n | n | n | n | n | n | n | n | n | n | n | n | n | n | n | n |  |  |  |  |
| *Nqua* | 1* | **+** | n | **+** | **+** | **+** | **+** | **+** | **-** | **+** | **+** | **+** | **+** | **+** | **-** | **+** | **+** | **+** | **+** | **-** | **-** | **+** | **-** |  |  |  |  |
|  | 2 | n | n | n | n | n | n | n | n | n | n | n | n | n | n | n | n | n | n | n | n | n | n |  |  |  |  |
|  | 3 | n | n | n | n | n | n | n | n | n | n | n | n | n | n | n | n | n | n | n | n | n | n |  |  |  |  |
| *Nsyl* | 1* | **+** | n | **+** | **-** | **-** | **+** | **+** | **-** | **+** | **+** | **+** | **+** | **+** | **-** | **+** | **+** | **+** | **+** | **-** | **+** | **+** | **+** |  |  |  |  |
|  | 2 | N | n | n | n | n | n | n | n | n | n | n | n | n | n | n | n | n | n | n | n | n | n |  |  |  |  |
|  | 3 | n | n | n | n | n | n | n | n | n | n | n | n | n | n | n | n | n | n | n | n | n | n |  |  |  |  |
| *Nvel* | 1* | **+** | n | **-** | **-** | **+** | **+** | **+** | **-** | **+** | **+** | **+** | **+** | **+** | **-** | **+** | **+** | **+** | **-** | **+** | **+** | **+** | **+** |  |  |  |  |
|  | 2 | n | n | n | n | n | n | n | n | n | n | n | n | n | n | n | n | n | n | n | n | n | n |  |  |  |  |
|  | 3 | n | n | n | n | n | n | n | n | n | n | n | n | n | n | n | n | n | n | n | n | n | n |  |  |  |  |
| *Same 1* | 1* | **+** | n | **+** | **-** | **-** | **+** | **+** | **-** | **+** | **-** | **+** | **-** | **+** | **-** | **-** | **-** | **+** | **-** | **+** | **+** | **+** | **+** |  |  |  |  |
|  | 2* | **+** | n | **+** | **+** | **+** | **+** | **+** | **-** | **+** | **+** | **+** | **+** | **+** | **+** | **+** | **+** | **+** | **+** | **+** | **+** | **+** | **+** |  |  |  |  |
|  | 3 | n | n | n | n | n | n | n | n | n | n | n | n | n | n | n | n | n | n | n | n | n | n |  |  |  |  |
|  | 4 | n | n | n | n | n | n | n | n | n | n | n | n | n | n | n | n | n | n | n | n | n | n |  |  |  |  |
| *Snig 3* | 1* | **+** | n | **+** | **+** | **+** | **+** | **+** | **+** | **+** | **+** | **+** | **+** | **+** | **+** | **+** | **+** | **+** | **+** | **+** | **+** | **+** | **+** |  |  |  |  |
|  | 2 | n | n | n | n | n | n | n | n | n | n | n | n | n | n | n | n | n | n | n | n | n | n |  |  |  |  |
|  | 3 | n | n | n | n | n | n | n | n | n | n | n | n | n | n | n | n | n | n | n | n | n | n |  |  |  |  |

a) Independent experiment. Experiment #1 is the same as shown in Tables 2 and 3 and Figure 3. In experiments indicated with an asterisk, five plants per line (10 spots analyzed) and in the other experiments three plants per line (6 spots analyzed) were inoculated. b) Plants were inoculated with *A. tumefaciens* mediating the expression of the T3Es indicated or GFP. Plant reactions were scored for 8 dpi and are represented by the color code described in Figure 1: red, strong necrosis (3 dpi); orange, weak necrosis (8 dpi); yellow, chlorosis (8 dpi); yellow/orange striped: chlorosis or cell death (8 dpi); white, no visible reaction (8 dpi). Colors were assigned if the same reaction was observed on ≥ 7/10 and ≥ 4/6 spots, respectively. Reactions on only 4-6/10 and 3/6 spots, respectively, were judged to be inconsistent, indicated in grey. T3E expression analyzed by immunoblot is indicated: +, expression detectable; -, no expression detectable; n, not analyzed. c) Plants were inoculated with *Xcv* 85-10, 85-10Δ*xopQ*, 85-10Δ*xopQ*(pBRM:xopQ) and 85-10Δ*hrcN* at OD_600_ = 0.4. The plant reactions are indicated by the same color code as used for *Agrobacterium*-mediated T3E expression, but symptoms were scored at 6 instead of 8 dpi. Green indicates water soaking. Crosses, not analyzed.
