# Supplementary material for: Non-host Resistance Induced by the Xanthomonas Effector XopQ Is Widespread within the Genus Nicotiana and Functionally Depends on EDS1
Source: Front Plant Sci. 2016 Nov 30;7:1796. doi: 10.3389/fpls.2016.01796 (PMC5127841; doi:10.3389/fpls.2016.01796)
Supplement: Supplementary file 6 [file Image1.PDF]

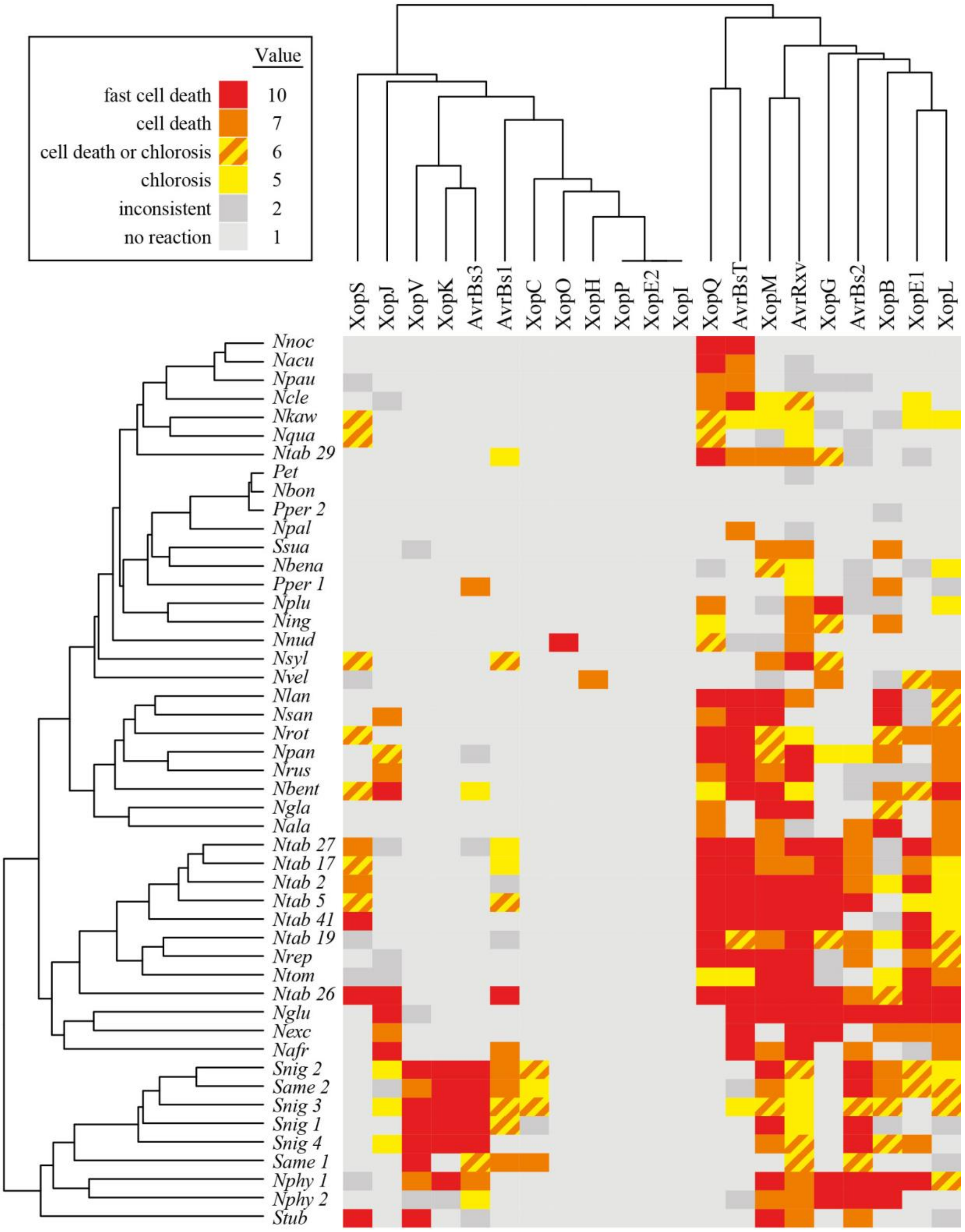

Supplementary Figure 1. Selected plant reactions to *Agrobacterium*-mediated transient expression of *Xcv* T3Es. Heatmap representation of effector responses in 48 different non-host *Solanaceae* plant lines (for abbreviations see Table S2). Five plants per line, two leaves per plant,

resulting in 10 spots per *Agrobacterium* strain, were inoculated with *Agrobacterium* strains mediating expression of the T3Es indicated on top. Plant reactions observed on at least 7/10 spots were classified as follows: fast cell death (3 dpi); cell death (6 dpi); chlorosis (6 dpi); chlorosis or cell death (6 dpi); no visible reaction (6 dpi). Reactions on only 4-6/10 spots were judged inconsistent. Plant reactions were visualized in a heatmap using the color code indicated. Each reaction type was assigned a value serving as the basis for clustering. The dendrogram shows the results of hierarchical clustering using average linkage and euklidean distance measures for T3Es and plant genotypes, respectively.
