# Supplementary figures and images for: Non-host Resistance Induced by the Xanthomonas Effector XopQ Is Widespread within the Genus Nicotiana and Functionally Depends on EDS1
Source: Front Plant Sci. 2016 Nov 30;7:1796. doi: 10.3389/fpls.2016.01796 (PMC5127841; doi:10.3389/fpls.2016.01796)

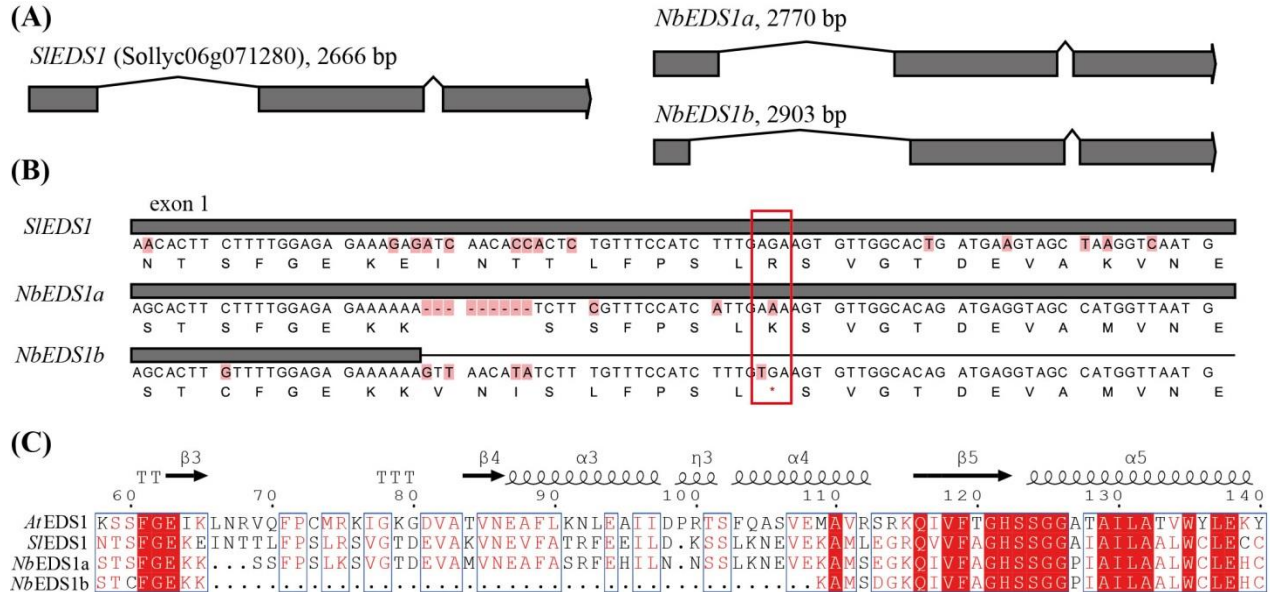

Supplement: Supplementary file 7 [file Image2.PDF]
